# Comparison of Risk Factors, Safety, and Efficacy Outcomes of Mechanical Thrombectomy in Posterior vs. Anterior Circulation Large Vessel Occlusion

**DOI:** 10.3389/fneur.2021.687134

**Published:** 2021-06-22

**Authors:** Joshua Mbroh, Khouloud Poli, Johannes Tünnerhoff, Alexandra Gomez-Exposito, Yi Wang, Benjamin Bender, Johann-Martin Hempel, Florian Hennersdorf, Katharina Feil, Annerose Mengel, Ulf Ziemann, Sven Poli

**Affiliations:** ^1^Department of Neurology & Stroke, Eberhard-Karls University, Tübingen, Germany; ^2^Hertie Institute for Clinical Brain Research, Eberhard-Karls University, Tübingen, Germany; ^3^Department of Neuroradiology, Eberhard-Karls University, Tübingen, Germany

**Keywords:** acute ischemic stroke, mechanical thrombectomy, endovascular stroke treatment, endovascular thrombectomy, posterior circulation, anterior circulation, large vessel occlusion, Mechanical Thrombectomy/Endovascular treatment, Ischemic Stroke, Large Vessel Occlusion

## Abstract

**Background and Purpose:** It is believed that stroke occurring due to posterior circulation large vessel occlusion (PCLVO) and that occurring due to anterior circulation large vessel occlusion (ACLVO) differ in terms of their pathophysiology and the outcome of their acute management in relation to endovascular mechanical thrombectomy (MT). Limited sample size and few randomized controlled trials (RCTs) with respect to PCLVO make the safety and efficacy of MT, which has been confirmed in ACLVO, difficult to assess in the posterior circulation. We therefore conducted a meta-analysis to study to which extent MT in PCLVO differs from ACLVO.

**Materials and Methods:** We searched the databases PubMed, Cochrane, and EMBASE for studies published between 2010 and January 2021, with information on risk factors, safety, and efficacy outcomes of MT in PCLVO vs. ACLVO and conducted a systematic review and meta-analysis; we compared baseline characteristics, reperfusion treatment profiles [including rates of intravenous thrombolysis (IVT) and onset-to-IVT and onset-to-groin puncture times], recanalization success [Thrombolysis In Cerebral Infarction scale (TICI) 2b/3], symptomatic intracranial hemorrhage (sICH), and favorable functional outcome [modified Rankin Score (mRS) 0–2] and mortality at 90 days.

**Results:** Sixteen studies with MT PCLVO (1,172 patients) and ACLVO (7,726 patients) were obtained from the search. The pooled estimates showed higher baseline National Institutes of Health Stroke Scale (NIHSS) score (SMD 0.32, 95% CI 0.15–0.48) in the PCLVO group. PCLVO patients received less often IVT (OR 0.65, 95% CI 0.53–0.79). Onset-to-IVT time (SMD 0.86, 95% CI 0.45–1.26) and onset-to-groin puncture time (SMD 0.59, 95% CI 0.33–0.85) were longer in the PCLVO group. The likelihood of obtaining successful recanalization and favorable functional outcome at 90 days was comparable between the two groups. PCLVO was, however, associated with less sICH (OR 0.56, 95% CI 0.37–0.85) but higher mortality (OR 1.92, 95% CI 1.46–2.53).

**Conclusions:** This meta-analysis indicates that MT in PCLVO may be comparably efficient in obtaining successful recanalization and 90 day favorable functional outcome just as in ACLVO. Less sICH in MT-treated PCLVO patients might be the result of the lower IVT rate in this group. Higher baseline NIHSS and longer onset-to-IVT and onset-to-groin puncture times may have contributed to a higher 90 day mortality in PCLVO patients.

## Introduction

Intravenous thrombolysis (IVT) has become the mainstay of acute intervention in ischemic stroke presenting within 4.5 h of symptom onset when other contraindications have been excluded (1). However, IVT has been shown to be less effective in proximal large vessel occlusion (LVO), mainly in the terminal internal carotid artery, proximal middle cerebral artery, and basilar artery, than in more distal occlusion (2). Therefore, clinical worsening is to be expected in many cases of LVO unless endovascular mechanical thrombectomy (MT) is initiated (2).

The second-generation MT devices that were introduced in the last decade have shown superiority to first-generation MT devices and, hence, have been widely used in MT since then (3–5). Consequently, it could be argued that most studies on MT conducted before the surge of second-generation MT devices could have been compromised by the inferiority of first-generation MT devices. Nowadays, MT in LVO may be conducted up to 24 h without waiting for IVT outcome (6–8).

To date, many randomized controlled trials (RCTs) have reported the safety and efficacy of MT in acute ischemic stroke due to anterior circulation LVO (ACLVO). However, there is lack of substantial data on the safety and efficacy of MT in posterior circulation LVO (PCLVO) (5, 6, 9).

Posterior circulation stroke is defined as the development of ischemic lesions occurring in the vascular territories supplied by branches of the vertebrobasilar arterial system (10). It occurs in about 20–25% of all ischemic strokes (11, 12), and neurological deficits caused by PCLVO have been described as catastrophic with severe disability and death occurring in about 68% of patients (13, 14). The rarity of PCLVO poses the challenge of obtaining a significant sample size for conducting observational and controlled trials in comparison to anterior circulation stroke.

MT in ACLVO has been accepted in most clinical settings as the best way for obtaining recanalization, and therefore, the randomization of patients with PCLVO into groups including no-MT is considered mostly unethical. Among the very few RCTs that focused on posterior circulation stroke, the Basilar Artery Occlusion Endovascular Intervention vs. Standard medical Treatment (BEST) RCT was terminated due to loss of equipoise, which resulted from a high crossover rate and was topped by a small sample size. This trial, however, reported no difference in favorable outcomes of MT patients and those receiving only standard medical treatment including IVT (15). On the other hand, a larger non-randomized cohort study, the Endovascular Treatment for Acute Basilar Artery Occlusion (BASILAR) study, reported that MT within 24 h of estimated occlusion time in basilar artery occlusion patients is associated with better functional outcomes and reduced mortality (16). Data on the safety and efficacy of MT in PCLVO from the randomized controlled Basilar Artery International Cooperation Study (BASICS) and Basilar Artery Occlusion Chines Endovascular Trial (BAOCHE) trials are pending (17, 18). Available data show a strong probability of differences in MT in PCLVO and ACLVO, which may contribute to their safety and efficacy outcomes (19, 20). An improved functional outcome and reduced mortality in moderate-to-severe ACLVO stroke patients have been shown to be dependent on a small infarct core, moderate-to-good collateral circulation, and rapid MT (21). Some PCLVO studies have associated MT with a poor outcome despite having a high recanalization rate, and this has raised interest in possible predicting factors of outcome in PCLVO such as initial stroke symptom severity, collateral status, age, infarct volume, stroke etiology, respiratory insufficiency, and other comorbidities (22–26). Bad outcome could also be a consequence of a delayed treatment since symptoms of posterior circulation stroke are known to be often fluctuating with about 55–63% cases of prodromal transient ischemic attack in spite of a persistent vessel occlusion (14).

Due to the conflicting nature of available studies, we conducted a systematic review and meta-analysis on studies published from 2010 to January 2021 with data comparing MT in PCLVO vs. ACLVO in order to assess the differences of risk factors, as well as safety and efficacy outcomes between both circulations.

## Materials and Methods

This study was conducted in accordance with the guidelines of the Preferred Reporting Item for Systematic Reviews and Meta-Analysis (PRISMA) (27).

### Data Source and Searching

We conducted a database search in PubMed, Cochrane library, and EMBASE before January 23, 2021, for literature from 2010 to 2021 using the following medical search heading (MeSH) and keywords: “acute stroke,” “mechanical thrombectomy,” “endovascular treatment,” “posterior circulation,” “vertebrobasilar occlusion,” “anterior circulation,” and “large vessel occlusion” (see Supplementary Methods). The PubMed search strategy was adapted for use in Cochrane library and EMBASE search databases. No restrictions were made in relation to literature type and text availability. Literature was however screened for study suitability based on title and abstract. Only subject-relevant studies were therefore assessed for eligibility.

### Study Selection and Data Extraction

Studies were included if they met the following criteria: (1) retrospective or prospective observational studies with a combined sample size for PCLVO and ACLVO of at least 40, (2) comparison of baseline characteristics and at least two reperfusion treatment profile parameters (i.e., rate of IVT, onset-to-IVT time, onset-to-groin puncture time, onset-to-recanalization time, and number of passages) in PCLVO and ACLVO as main and/or subgroup analysis, and (3) outcome defined by at least two of the following: Thrombolysis In Cerebral Infarction scale (TICI) 2b/3 (28), symptomatic intracranial hemorrhage (sICH), modified Rankin Scale score (mRS) 0–2 at 90 days, and mortality at 90 days. Exclusion criteria included the following: (1) non-English literature, (2) no MT conducted, (3) duplicate literature, (4) insufficient data for comparison purposes, and (5) same datasets used by multiple studies. Duplicates were identified and eliminated using EndNote X9 citation manager software (Clarivate, Philadelphia, PA, USA).

Data extracted from the included studies were patient age, sex, comorbidities/cardiovascular risk factors, stroke etiology, baseline NIHSS, site of LVO, reperfusion treatment profile (rate of IVT, onset-to-IVT time, onset-to-groin puncture time, onset-to-recanalization time, and number of passages), TICI 2b/3, sICH, 90 day mRS 0–2 and mortality.

### Quality Assessment

Quality assessment by means of Risk of Bias Assessment tool for Non-randomized Studies (RoBANS) was performed to assess the methodological quality of all included studies under which studies were rated as having either a high, a low, or an uncertain risk of bias (29).

Data search, eligibility assessment, selection, and extraction as well as quality assessment were conducted and crosschecked by two independent investigators and contentions were resolved through a consensus between the two. Publication bias was assessed by means of a funnel plot asymmetry.

### Statistical Analysis

Statistical analyses were performed using Review Manager (RevMan) [Computer program], version 5.4.1 (30). We used the Mantel–Haenszel statistical method and a random or fixed effects analysis model for studies with moderate/high and low heterogeneity, respectively, to estimate the pooled effect size. Cochrane *I*^2^ statistics was used to assess heterogeneity. We defined high heterogeneity as *I*^2^ > 75%, moderate heterogeneity as 25% < *I*^2^ <75%, and low heterogeneity as *I*^2^ <25% (31). PCLVO and ACLVO were the comparison groups, and the corresponding meta-analysis was performed for each outcome of interest.

Sensitivity analysis was performed for baseline characteristics and recanalization treatment profiles in which retrospective studies were excluded due to higher risk of selection bias (29). We furthermore conducted subgroup analyses in which we excluded studies with <20 patients with PCLVO due to a possible risk of lack of representation of PCLVO with low sample sizes in the real world and studies that primarily recruited patients who received MT until 2012, with the presumption that results could have been compromised by the use of first-generation MT devices (32).

Odds ratios (ORs) and standardized mean difference (SMD) were calculated with 95% confidence intervals (CIs) and *p* < 0.05 was considered significant. For continuous data, the corresponding estimated mean and estimated standard deviation were calculated (33).

## Results

### Search Results and Study Characteristics

**Figure 1:**
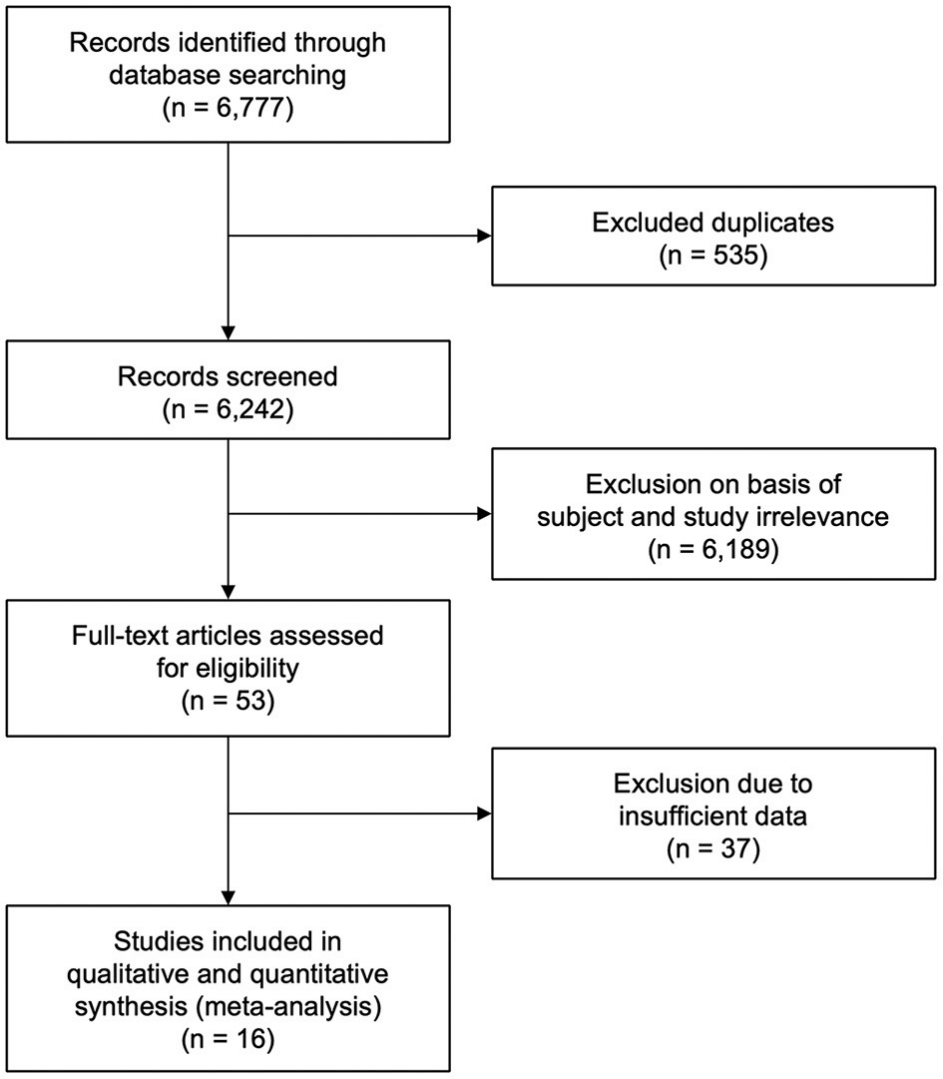
Preferred Reporting Item for Systematic Reviews and Meta-Analysis (PRISMA) flow diagram showing study selection (27).

The database search yielded 6,777 citations of literature published between January 1, 2010 and January 23, 2021. A total of 535 duplicate studies were excluded. A total of 6,242 studies were screened, out of which 6,189 were eliminated on the basis of subject and study irrelevance. The remaining 53 studies were individually assessed for eligibility by means of full-text review with 37 studies being excluded due to lack of sufficient data. Sixteen eligible studies that met the study inclusion criteria were therefore included in our meta-analysis (Figure 1). Data from 11 studies (26, 34–43) were acquired from multiple centers and data from 5 studies (44–48) were acquired from single centers. Among these studies, 6 were retrospective (35, 39, 44–47) and 10 were prospective studies (26, 34, 36–38, 40–43, 48). This study comprised a total of 8,898 patients with 1,172 belonging to PCLVO and 7,726 belonging to ACLVO.

### Risk of Bias Assessment

Data on risk of bias are shown in Supplementary Figure 1; generally, there was a high risk of bias with respect to patient selection, confounding variables, and outcome reporting and a lower risk of incomplete data across all studies.

The funnel plots showed no asymmetry with respect to sICH, indicating a low probability of publication bias across the included studies for sICH. Asymmetry was, however, observed for recanalization success, favorable functional outcome, and mortality (Supplementary Figure 2).

### Baseline Characteristics

**Figure 2:**
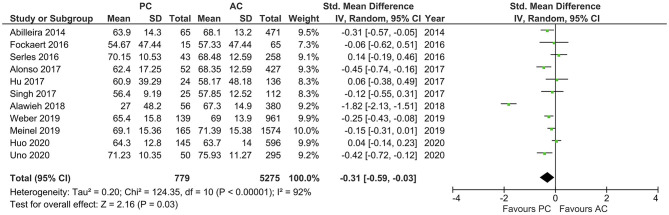
Forest plot comparing “age” of patients with large vessel occlusion in the posterior circulation (PC) vs. anterior circulation (AC) who were treated with endovascular mechanical thrombectomy. Chi^2^, chi-square statistic; CI, confidence interval; df, degrees of freedom; *I*^2^, *I*-square heterogeneity statistic; IV, weighted mean difference; *P, p*-value; SD, standard deviation; Std., standardized; Tau^2^, estimated variance of underlying effects across studies; *Z, Z* statistic.

**Figure 3:**
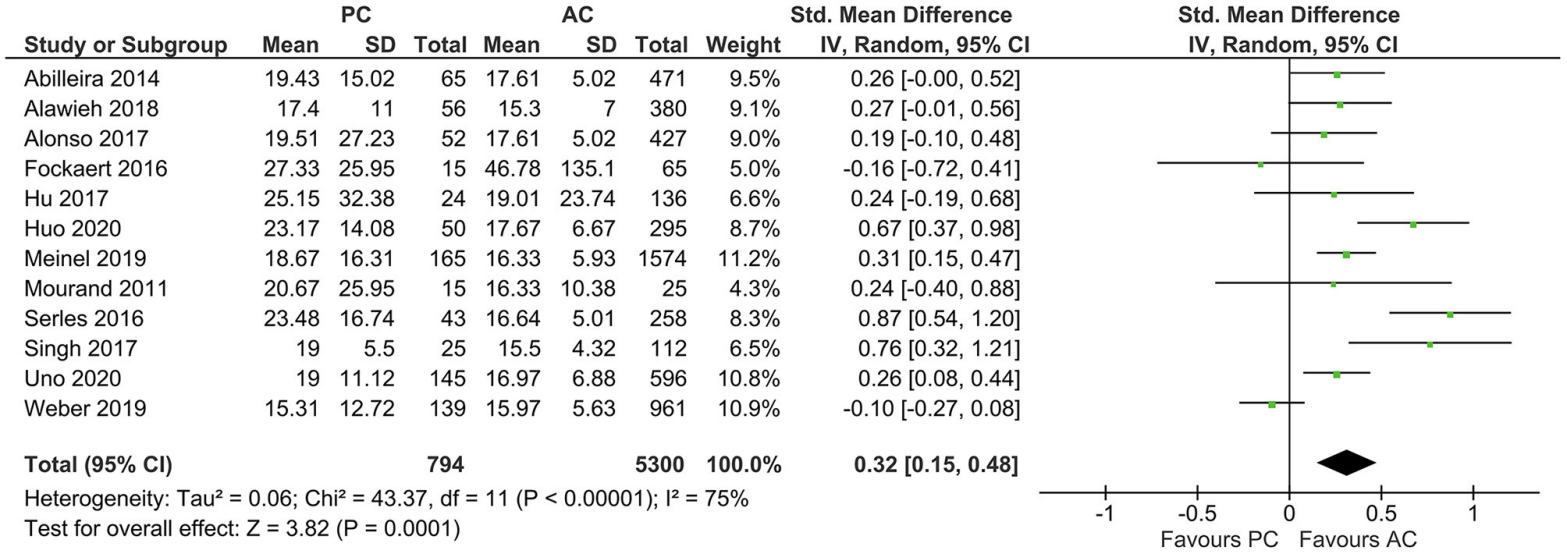
Forest plot comparing the baseline characteristic “female sex” of patients with large vessel occlusion in the posterior circulation (PC) vs. anterior circulation (AC) who were treated with endovascular mechanical thrombectomy. Chi^2^, chi-square statistic; CI, confidence interval; df, degrees of freedom; *I*^2^, *I*-square heterogeneity statistic; M-H, Mantel–Haenszel statistic; *P, p*-value; Tau^2^, estimated variance of underlying effects across studies; *Z, Z* statistic.

Results of age, sex, baseline NIHSS, and site of LVO for the included studies are reported in Table 1. PCLVO patients were younger than ACLVO [SMD = −0.31 (95% CI 0.59–0.03), *p* = 0.03] (*I*^2^ = 92%, *p* < 0.00001) (Figure 2). Further results showed less females in the PCLVO group [OR = 0.54 (95% CI 0.39–0.73), *p* < 0.0001] (*I*^2^ = 59%, *p* = 0.008) (Figure 3).

**Table 1:** Baseline characteristics reported in included studies.

	**Posterior circulation**					**Anterior circulation**				
**Publication**	***N***	**Age, years**	**Female sex**	**Baseline NIHSS**	**Occluded vessel**	***N***	**Age, years**	**Female sex**	**Baseline NIHSS**	**Occluded vessel**
Mourand et al. (44)	15	–	–	21 (3–38)	14 BA, 1 VA	25	–	–	17 (9–23)	16 MCA, 9 ICA
Abilleira et al. (34)	65	64 ± 14	21	16 (8–27)	–	471	68 ± 13	221	18 (14–21)	–
Lefevre et al. (45)	26	–	–	–	25 BA, 1 PCA	36	–	–	–	20 ICA, 16 MCA
Fockaert et al. (35)	15	56 (22–86)	2	33 (7–42)	15 PCA	65	64 (22–86)	43	15 (6–42)	47 MCA, 18 ICA
Serles et al. (36)	43	72 (63–77)	19	19 (13–30)	40 BA, 3 VA	258	70 (60–77)	133	17 (13–20)	189 MCA, 65 ICA, 1 ACA
Alonso De Lecinana et al. (26)	52	64 (50–74)	17	11 (6–23)	52 VBA	427	70 (60–77)	214	18 (14–21)	284 MCA, 100 ICA, 43 tandem occlusion
Hu et al. (46)	24	66 (32–85)	11	14 (2–34)	24 VBA	137	66 (22–87)	59	10 (3–26)	94 MCA, 42 ICA, 1 ACA
Khoury et al. (37)	5	–	–	–	5 VBA	35	–	–	–	29 MCA, 6 ICA
Singh et al. (38)	25	56 ± 9	9	19 ± 9	25 BA	112	58 ± 13	41	16 ± 13	61 MCA, 51 ICA
Alawieh et al. (47)	56	27 ± 48	8	17 ± 11	–	380	67 ± 15	192	15 ± 7	–
Meinel et al. (39)	165	70 (59–80)	69	18 (8–30)^*^	–	1,574	73 (61–82)	810	17 (12–20)^**^	–
Weber et al. (40)	139	65 ± 16	–	12 (6–21)	–	961	69 ± 14	–	15 (12–19)	–
Wollenweber et al. (41)	303	–	–	–	250 BA, 69 PCA, 59 VA	2,265	–	–	–	1,890 MCA, 666 ICA, 86 ACA
Uno et al. (48)	50	73 (65–79)	17	25 (13–32)	–	295	77 (69–84)	151	18 (13–22)	–
Renieri et al. (43)	44	–	–	–	38 BA, 4 VA, 2 PCA	90	–	–	–	53 MCA, 37 ICA
Huo et al. (42)	145	64 ± 13	37	20 (11–26)	–	596	64 ± 14	216	16 (12–21)	–

* *n = 155*,

** *n = 1,558; – = not available, ACA, anterior cerebral artery; BA, basilar artery; ICA, internal carotid artery; MCA, middle cerebral artery; N, number of patients; n, reference number of patients; NIHSS, National Institutes of Health Stroke Scale score; PCA, posterior cerebral artery; VA, vertebral artery; VBA, vertebrobasilar arteries*.

Hypertension seemed to be the predominant comorbidity in both PCLCO (56.0%) and ACLVO (62.6%) (Supplementary Table 1). Although the pooled results showed hypertension to be comparable in both circulations, there seemed to be a tendency of fewer cases of hypertension as a comorbidity in PCLVO [OR = 0.76 (95% CI 0.54–1.09), *p* = 0.14] (*I*^2^ = 76%, *p* < 0.0001) (Supplementary Figure 3). In addition, atrial fibrillation [OR = 0.62 (95% CI 0.50–0.77), *p* < 0.00001] (*I*^2^ = 0%, *p* = 0.71) and hyperlipidemia [OR = 0.73 (95% CI 0.61–0.89), *p* = 0.001] (*I*^2^ = 9%, *p* = 0.36) were less likely comorbidities of PCLVO, with smoking being a more likely comorbidity of PCLVO [OR = 1.22 (95% CI 1.01–1.48), *p* = 0.004] (*I*^2^ = 0%, *p* = 0.47) (Supplementary Figures 4–6, respectively). Diabetes mellitus [OR = 0.98 (95% CI 0.72–1.34), *p* = 0.91] (*I*^2^ = 51%, *p* = 0.04), coronary artery disease [OR = 0.64 (95% CI 0.36–1.27), *p* = 0.22] (*I*^2^ = 62%, *p* = 0.02), and previous stroke/TIA [OR = 1.21 (95% CI 0.96–1.53), *p* = 0.11] (*I*^2^ = 17%, *p* = 0.30) were, however, comparable between both groups (Supplementary Figures 7–9, respectively).

**Figure 4:**
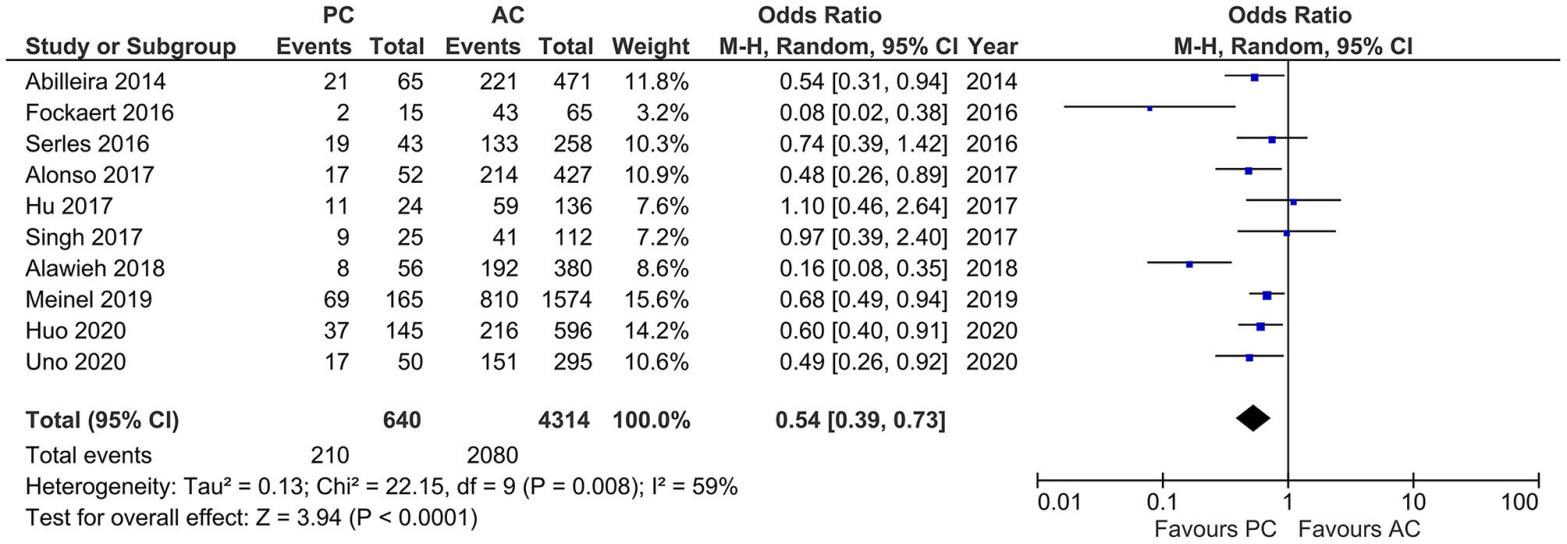
Forest plot comparing “admission NIHSS” of patients with large vessel occlusion in the posterior circulation (PC) vs. anterior circulation (AC) who were treated with endovascular mechanical thrombectomy. Chi^2^, chi-square statistic; CI, confidence interval; df, degrees of freedom; *I*^2^, *I*-square heterogeneity statistic; IV, weighted mean difference; *P, p*-value; SD, standard deviation; Std., standardized; Tau^2^, estimated variance of underlying effects across studies; *Z, Z* statistic.

The average baseline NIHSS was higher in PCLVO [SMD = 0.32 (95% CI 0.15–0.48), *p* = 0.0001] (*I*^2^ = 75%, *p* < 0.00001) (Figure 4). In ACLVO, middle cerebral artery occlusion was the most prevalent site of LVO (31.0%) followed by internal carotid artery (13.1%). Basilar artery occlusion was the predominant lesion location in the PCLVO (33.5%) followed by posterior cerebral artery (7.4%).

Large artery atherosclerosis was a more likely stroke etiology in PCLVO [OR = 1.55 (95% CI 1.26–1.91), *p* < 0.0001] (*I*^2^ = 0%, *p* = 0.76) in comparison to ACLVO (Supplementary Figure 10). On the other hand, cardiac embolism was a less likely stroke etiology in PCLVO [OR = 0.63 (95% CI 0.52–0.75), *p* < 0.0001] (*I*^2^ = 0%, *p* = 0.67) in comparison to ACLVO (Supplementary Figure 11). Results obtained from sensitivity analyses conducted for the baseline characteristics age, sex, admission NIHSS, stroke etiology, and all comorbidities except “prior stroke or transient ischemic attack” had no influence on their respective results (Supplementary Figures 3–8, 10–14). However, sensitivity analysis showed “prior stroke or transient ischemic attack” being more likely in PCLVO [OR = 1.39 (95% CI 1.06–1.82), *p* = 0.02] (*I*^2^ = 0%, *p* = 0.51) (Supplementary Figure 9).

### Recanalization Treatment Profiles

**Figure 5:**
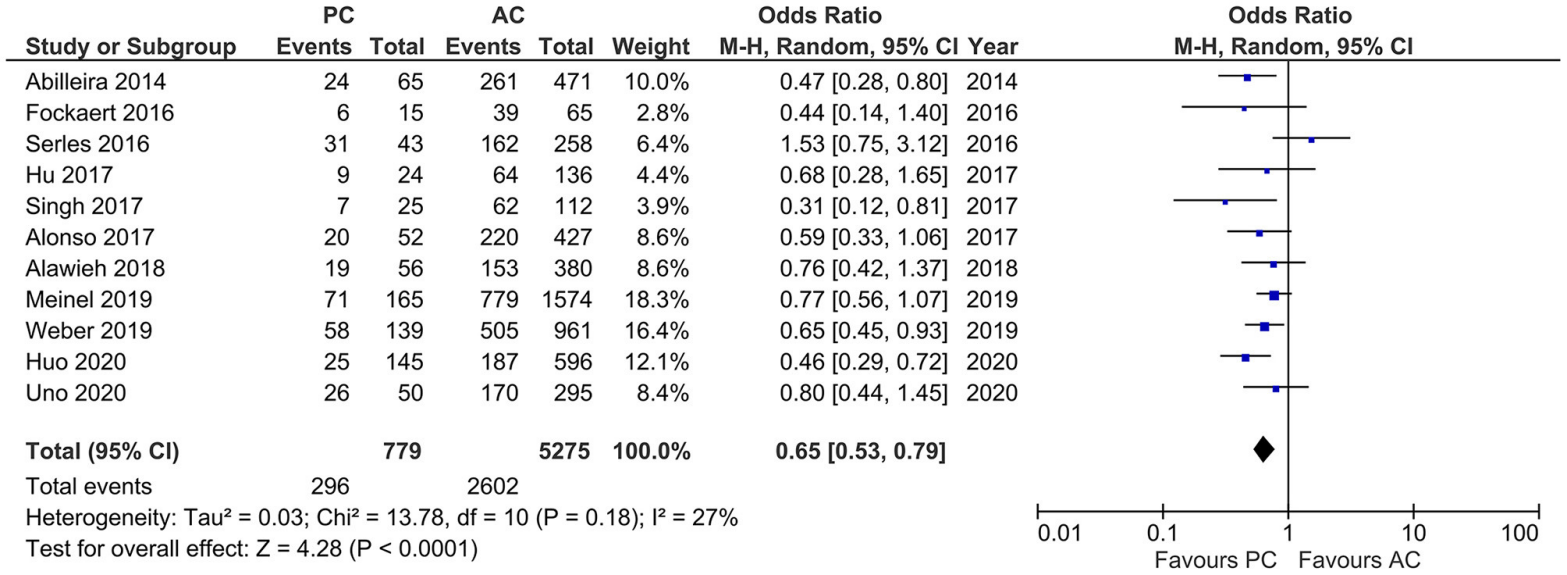
Forest plot comparing rate of intravenous thrombolysis in patients with large vessel occlusion in the posterior circulation (PC) vs. anterior circulation (AC) who were treated with endovascular mechanical thrombectomy. Chi^2^, chi-square statistic; CI, confidence interval; df, degrees of freedom; *I*^2^, *I*-square heterogeneity statistic; M-H, Mantel–Haenszel statistic; *P, p*-value; Tau^2^, estimated variance of underlying effects across studies; *Z, Z* statistic.

Studies that reported number of IVT showed moderate heterogeneity (*I*^2^ = 27%, *p* < 0.0001). The pooled results indicated a lower frequency of IVT in PCLVO patients [OR = 0.65 (95% CI 0.53–0.79), *p* < 0.0001] (Figure 5).

**Figure 6:**
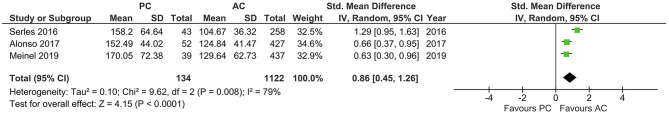
Forest plot comparing “onset-to-intravenous thrombolysis time” in patients with large vessel occlusion in the posterior circulation (PC) vs. anterior circulation (AC) who were treated with endovascular mechanical thrombectomy. Chi^2^, chi-square statistic; CI, confidence interval; df, degrees of freedom; *I*^2^, *I*-square heterogeneity statistic; IV, weighted mean difference; *P, p*-value; SD, standard deviation; Std., standardized; Tau^2^, estimated variance of underlying effects across studies; *Z, Z* statistic.

**Figure 7:**
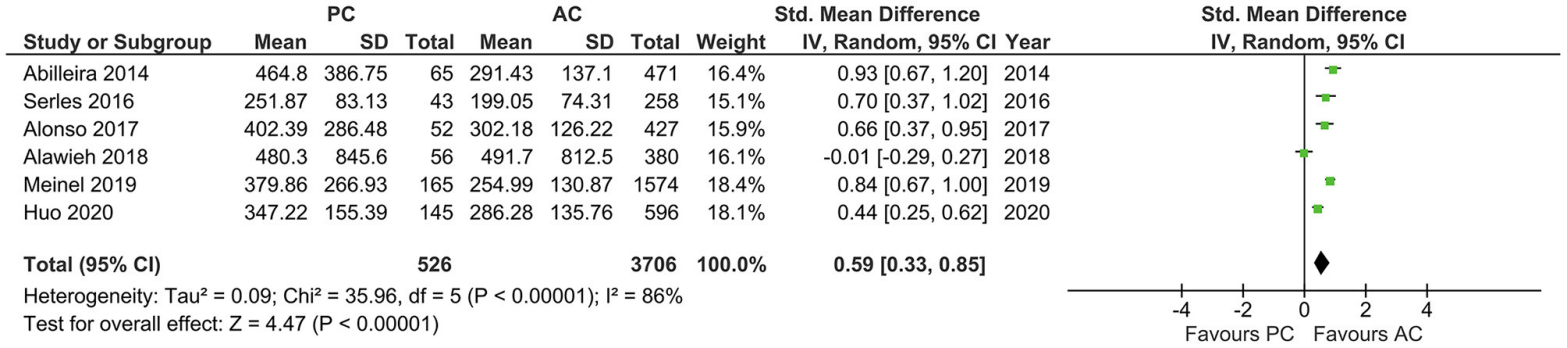
Forest plot comparing “onset-to-groin puncture time” in patients with large vessel occlusion in the posterior circulation (PC) vs. anterior circulation (AC) who were treated with endovascular mechanical thrombectomy. Chi^2^, chi-square statistic; CI, confidence interval; df, degrees of freedom; *I*^2^, *I*-square heterogeneity statistic; IV, weighted mean difference; *P, p*-value; SD, standard deviation; Std., standardized; Tau^2^, estimated variance of underlying effects across studies; *Z, Z* statistic.

With a high heterogeneity across the studies reporting onset to IVT (*I*^2^ = 79%, *p* = 0.008), the pooled estimates showed a longer onset to IVT in PCLVO [SMD = 0.86 (95% CI 0.45–1.26), *p* < 0.0001] (Figure 6). Further analyses also showed a longer onset-to-groin puncture time in PCLVO [SMD = 0.59 (95% CI 0.33–0.85), *p* < 0.00001] (*I*^2^ = 86%, *p* < 0.00001) (Figure 7). Results from onset-to-recanalization time in PCLVO, however, did not show any difference compared to ACLVO [SMD = 0.29 (95% CI −0.04–0.60), *p* = 0.08] (*I*^2^ = 90%, *p* < 0.00001) (Supplementary Figure 15). Pooled results for the number of passages did not reveal a difference between PCLVO and ACLVO [SMD = 0.21 (95% CI −0.05–0.46), *p* = 0.11] (*I*^2^ = 79%, *p* = 0.0008) (Supplementary Figure 16).

Sensitivity analyses performed for rates of IVT, onset-to-IVT time, onset-to-groin puncture time, and number of passages had no influence on their respective results (Supplementary Figures 16–19). However, sensitivity analysis showed PCLVO to be associated with a longer onset-to-recanalization time [SMD = 0.43 (95% CI 0.10–0.77), *p* = 0.01] (*I*^2^ = 89%, *p* < 0.00001) (Supplementary Figure 15).

### Outcomes of Study

Results of TICI 2b/3, sICH, mRS 0-2 at 90 days, and mortality for the included studies are reported in Table 2.

**Table 2:** Outcomes reported in included studies.

	**Posterior circulation**					**Anterior circulation**				
**Publication**	***N***	**Successful recanalization**	**sICH**	**mRS 0–2 at 90 days**	**Mortality**	***N***	**Successful recanalization**	**sICH**	**mRS 0–2 at 90 days**	**Mortality**
Mourand et al. (44)	15	11	–	5^*^	7	25	15	–	9	8^‡^
Abilleira et al. (34)	65	48	5	25	22	471	348	25	207	97
Lefevre et al. (45)	26	23	–	14	–	36	23	–	11	–
Fockaert et al. (35)	15	15	2	–	7	65	47	2	–	14
Serles et al. (36)	43	35	0	–	10	258	207	18	–	24
Alonso De Lecinana et al. (26)	52	39	1	21	17	427	359	23	237	48
Hu et al. (46)	24	19	1	–	4	137	110	12	–	8
Khoury et al. (37)	5	–	–	1	4	35	–	–	19	7
Singh et al. (38)	25	21	–	–	2	112	104	–	–	7
Alawieh et al. (47)	56	54	3	24	16	380	351	20	164	68
Meinel et al. (39)	165	149	8	55^**^	55^**^	1,574	1,299^‡‡^	98^‡‡‡^	604^§§^	344^§§^
Weber et al. (40)	139	96^#^	0	35^***^	31^***^	961	719^§^	29	281^§§§^	203^§§§^
Wollenweber et al. (41)	303	246	–	100^##^	82^##^	2,265	1,857	–	732^†^	570^†^
Uno et al. (48)	50	50	0	27^###^	4^###^	295	250	38^†††^	105^††^	22^††^
Renieri et al. (43)	44	33	–	–	–	90	65	–	–	–
Huo et al. (42)	145	119	4	–	49	596	520	44	–	98

* *n = 14*,

** *n = 152*,

*** *n = 92*,

# *n = 134*,

## *n = 265*,

### *n = 42*,

‡ *n = 22*,

‡‡ *n = 1571*,

‡‡‡ *n = 1,562*,

§ *n = 964*,

§§ *n = 1,409*,

§§§ *n = 660*,

† *n = 1,997*,

†‡ *n = 213*,

‡†† *n = 290; – = not available, sICH, symptomatic intracranial hemorrhage; mRS, modified ranking score; N, number of patients; n, reference number of patients*.

#### Successful Recanalization

**Figure 8:**
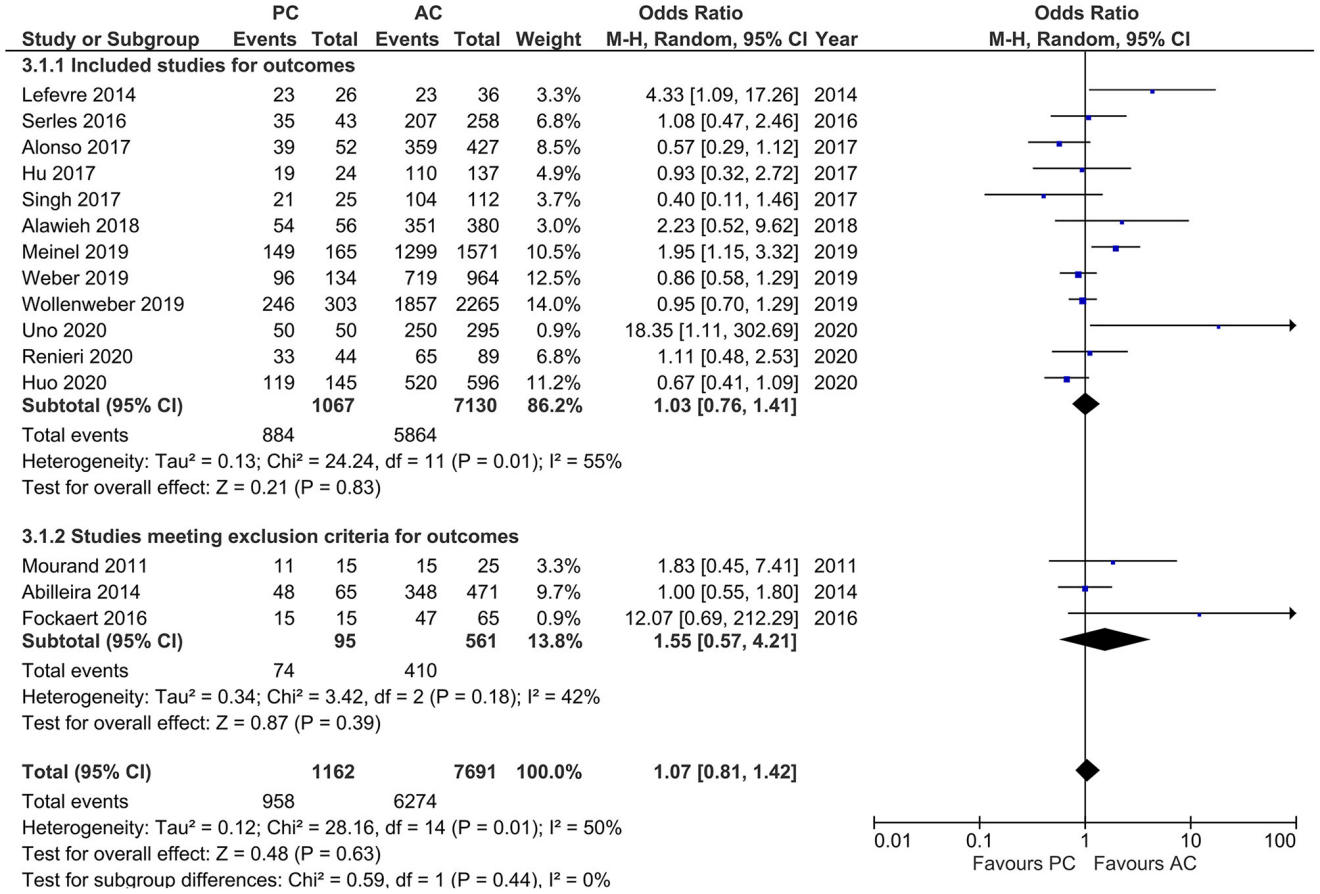
Forest plot of main and subgroup analyses comparing “successful recanalization” defined as thrombolysis in cerebral infarction scale (TICI) 2/3 in patients with large vessel occlusion (LVO) in the posterior circulation (PC) vs. anterior circulation (AC) who were treated with endovascular mechanical thrombectomy. Three studies with <20 PCLVO patients and/or patient recruitment primarily until 2012 were excluded from subgroup analysis. Chi^2^, chi-square statistic; CI, confidence interval; df, degrees of freedom; *I*^2^, *I*-square heterogeneity statistic; M-H, Mantel–Haenszel statistic; *P, p*-value; Tau^2^, estimated variance of underlying effects across studies; *Z, Z* statistic.

Studies that reported successful recanalization (i.e., TICI 2b/3) showed moderate heterogeneity (*I*^2^ = 50%, *p* = 0.01). The pooled estimates showed no difference in outcomes in both PCLVO and ACLVO [OR = 1.07 (95% CI 0.81–1.42), *p* = 0.44]. In a subgroup analysis in which three studies were excluded on the basis of <20 PCLVO patients and patient recruitment primarily until 2012, the remaining studies showed similar results [OR = 1.03 (95% CI 0.76–1.41), *p* = 0.83] (*I*^2^ = 55%, *p* = 0.01) (Figure 8).

#### Symptomatic Intracerebral Hemorrhage

**Figure 9:**
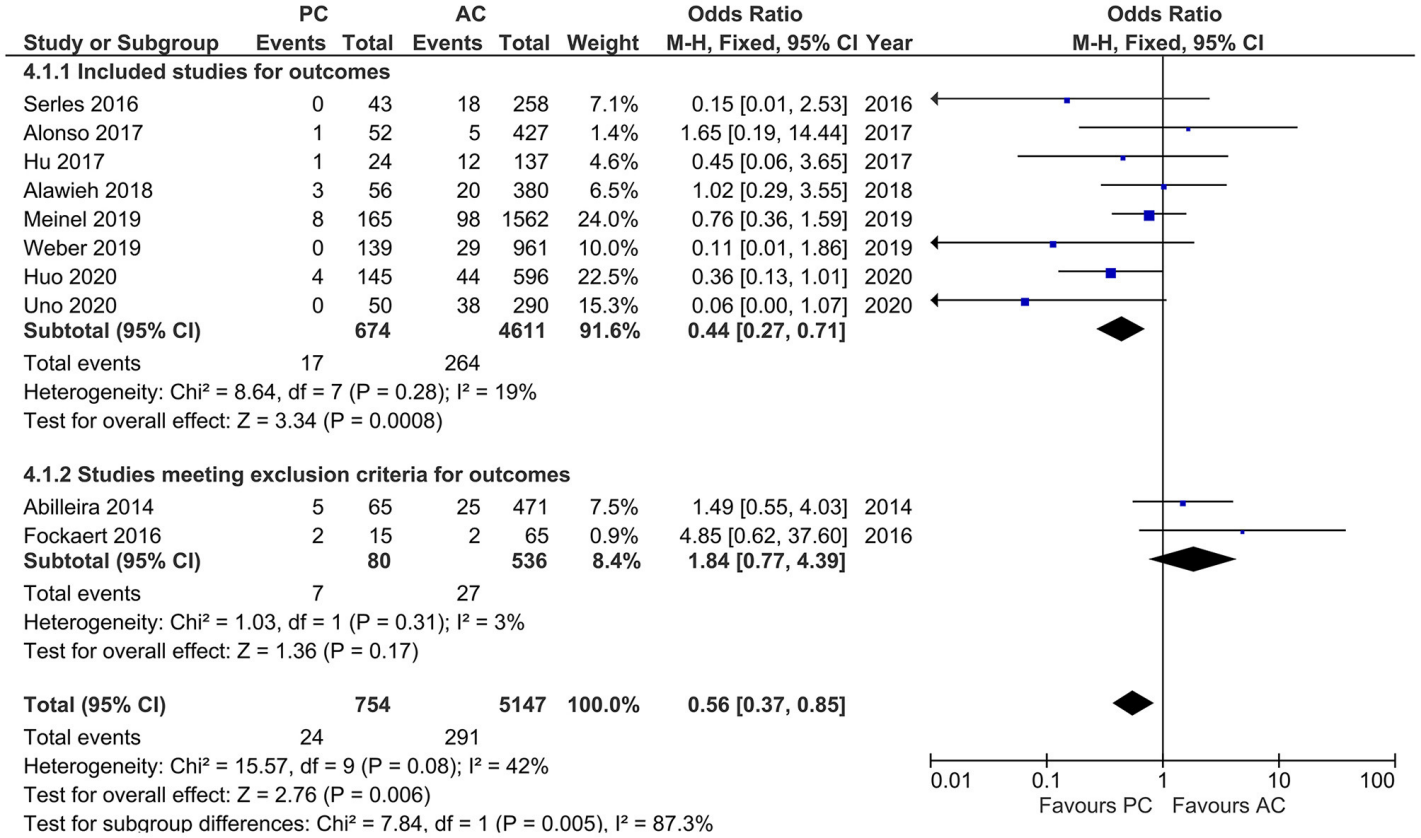
Forest plot comparing “symptomatic intracranial hemorrhage” in patients with large vessel occlusion (LVO) in the posterior circulation (PC) vs. anterior circulation (AC) who were treated with endovascular mechanical thrombectomy. Two studies with <20 PCLVO patients and/or patient recruitment primarily until 2012 were excluded from subgroup analysis. Chi^2^, chi-square statistic; CI, confidence interval; df, degrees of freedom; *I*^2^, *I*-square heterogeneity statistic; M-H, Mantel–Haenszel statistic; *P, p*-value; *Z, Z* statistic.

Studies that reported sICH showed moderate heterogeneity (*I*^2^ = 42%, *p* = 0.08). Our results indicated a lower likelihood of sICH in PCLVO [OR = 0.56 (95% CI 0.37–0.85), *p* = 0.006]. In our subgroup analysis (exclusion of two studies based on <20 PCLVO patients and patient recruitment primarily until 2012), the studies showed a rather reduced heterogeneity (*I*^2^ = 19%, *p* = 0.28). The pooled estimates once again indicated a lower likelihood of sICH in PCLVO compared to ACLVO [OR = 0.44 (95% CI 0.27–0.71), *p* = 0.0008] (Figure 9).

#### Favorable Functional Outcome at 90 Days

**Figure 10:**
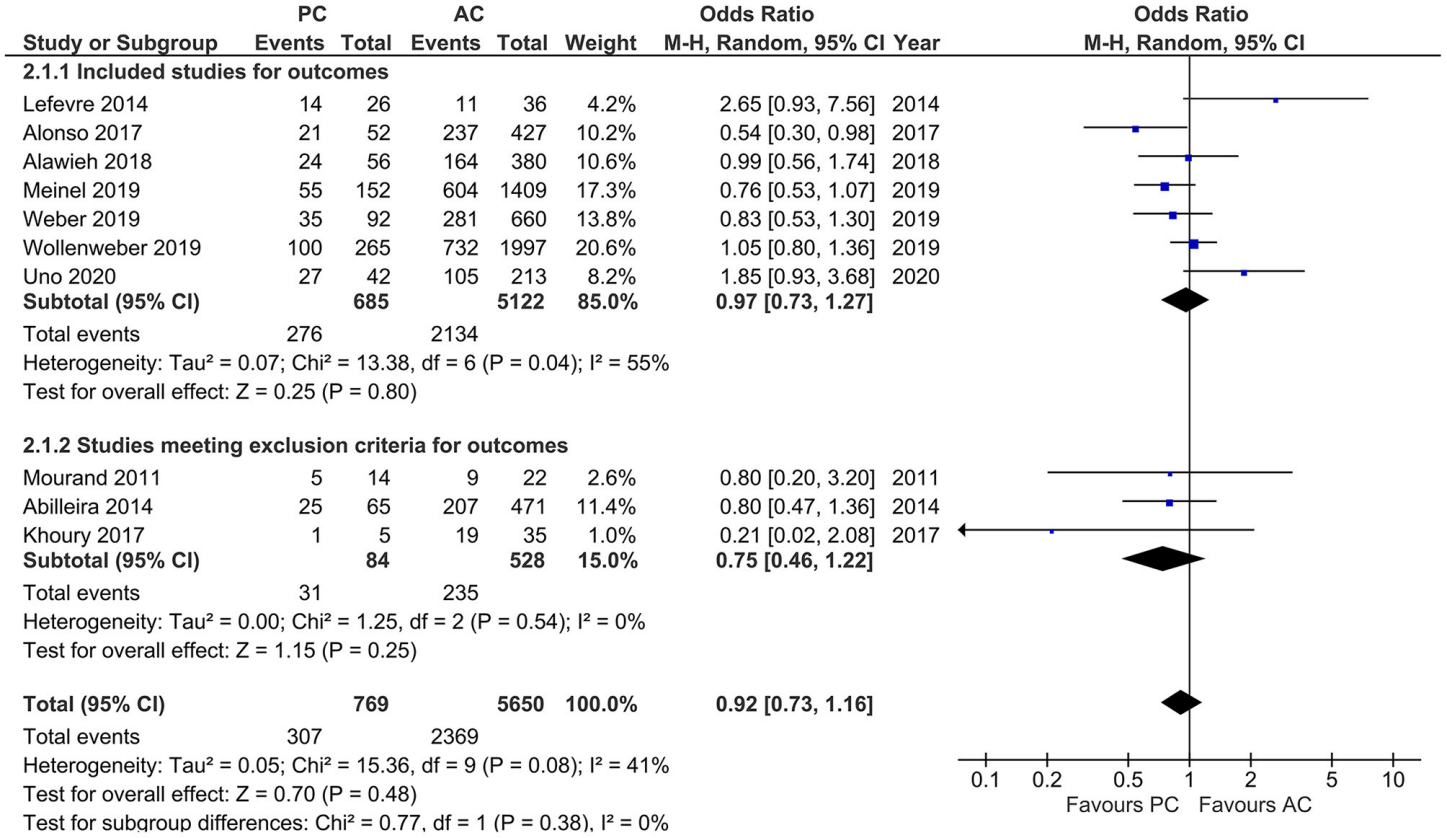
Forest plot comparing “favorable functional outcome (defined as modified Rankin Scale score 0–2) at 90 days” in patients with large vessel occlusion (LVO) in the posterior circulation (PC) vs. anterior circulation (AC) who were treated with endovascular mechanical thrombectomy. Three studies with <20 PCLVO patients and/or patient recruitment primarily until 2012 were excluded from subgroup analysis. Chi^2^, chi-square statistic; CI, confidence interval; df, degrees of freedom; *I*^2^, *I*-square heterogeneity statistic; M-H, Mantel–Haenszel statistic; *P, p*-value; Tau^2^, estimated variance of underlying effects across studies; *Z, Z* statistic.

Studies that reported favorable functional outcome, defined by mRS ≤ 2 at 90 days, showed a moderate heterogeneity (*I*^2^ = 41%, *p* = 0.08) with comparable likelihood of favorable functional outcome in both PCLVO and ACLVO [OR = 0.92 (95% CI 0.73–1.16), *p* = 0.48]. The subgroup analysis (after exclusion of three studies based on <20 PCLVO patients and patient recruitment primarily until 2012) showed similar findings (*I*^2^ = 55%, *p* = 0.04) [OR = 0.97 (95% CI 0.73–1.27), *p* = 0.80] (Figure 10).

#### Mortality

**Figure 11:**
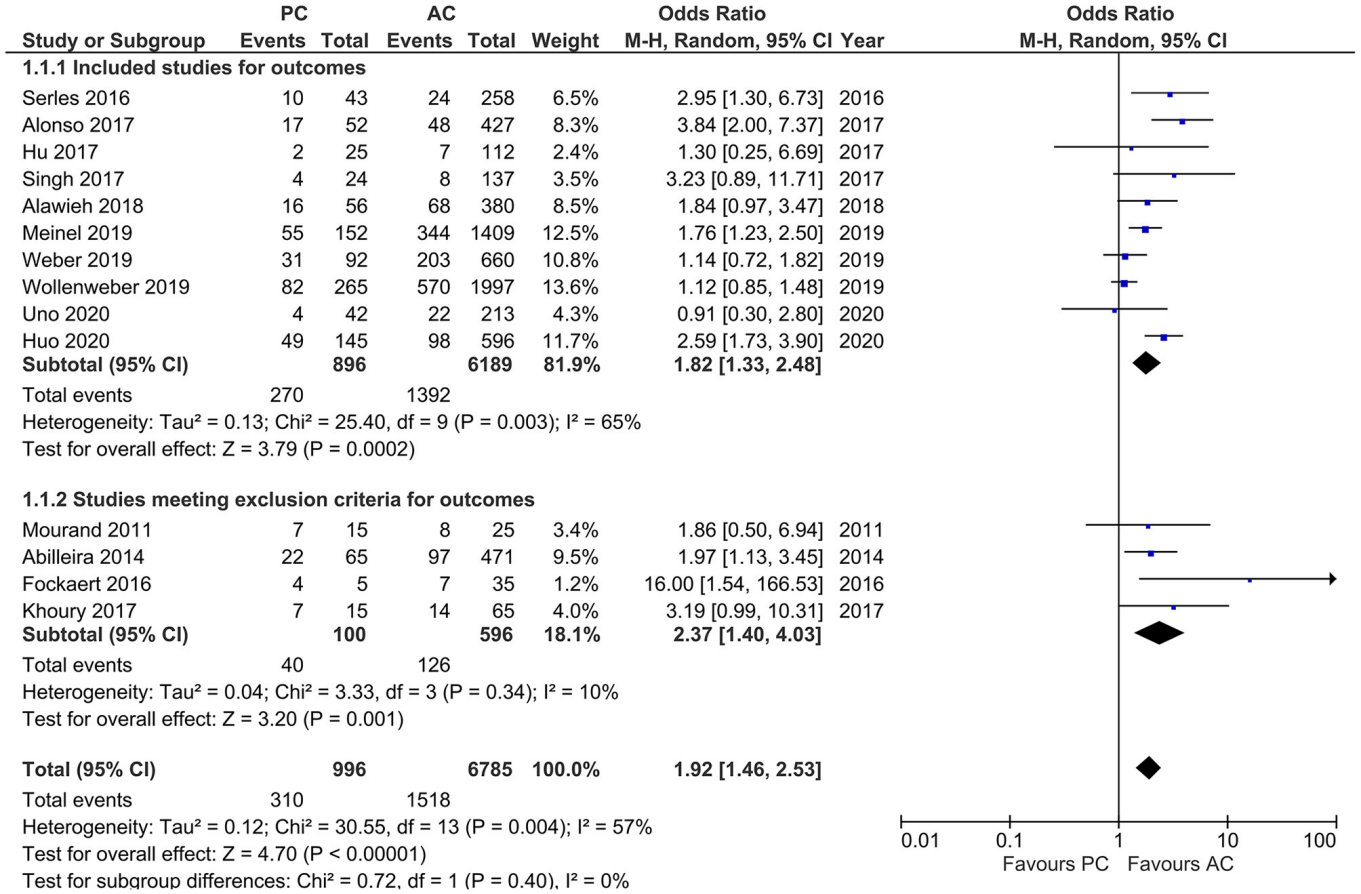
Forest plot comparing “mortality at 90 days” in patients with large vessel occlusion (LVO) in the posterior circulation (PC) vs. anterior circulation (AC) who were treated with endovascular mechanical thrombectomy. Four studies with <20 PCLVO patients and/or patient recruitment primarily until 2012 were excluded from subgroup analysis. Chi^2^, chi-square statistic; CI, confidence interval; df, degrees of freedom; *I*^2^, *I*-square heterogeneity statistic; M-H, Mantel–Haenszel statistic; *P, p*-value; Tau^2^, estimated variance of underlying effects across studies; *Z, Z* statistic.

Studies reporting mortality showed a moderate heterogeneity (*I*^2^ = 57%, *p* = 0.004). MT in PCLVO was associated with a higher likelihood of mortality as compared to ACLVO [OR = 1.92 (95% CI 1.46–2.53), *p* < 0.00001]. The subgroup analysis (after exclusion of four studies due to <20 PCLVO patients and patient recruitment primarily until 2012) likewise showed a higher likelihood of mortality in PCLVO patients [OR = 1.82 (95% CI 1.33–2.48), *p* = 0.0002] (*I*^2^ = 65%, *p* = 0.003) (Figure 11).

## Discussion

To the best of our knowledge, until the conduction of this meta-analysis, there had been two prior meta-analyses comparing MT in anterior and posterior circulation stroke with both studies, however, focusing on MT safety and efficacy outcomes (19, 20). This study, conducted independently from previous studies, included more recent literature on MT in PCLVO and ACLVO and, in a detailed meta-analysis, further sought to compare demographics and baseline characteristics, risk factors, as well as recanalization treatment profiles between the two brain circulations. Hence, this study presents at the time of publication the most current data on MT in PCLVO vs. ACLVO.

Results on etiology of LVO in our meta-analysis show large artery atherosclerosis (36.7% and 23.1%) and cardiac embolism (34.8 and 47.0%) to be the most common causes of PCLVO and ACLVO, respectively (Supplementary Table 2), with large artery atherosclerosis and cardiac embolism being an equally likely etiology in PCLVO. This was consistent with reports from previous literature that reported 26–36% for large artery atherosclerosis and 30–35% for cardiac embolism in PCLVO, although this was based only on basilar artery occlusion (14). The difference in stroke etiologies for the other classification groups such as other determined LVO etiologies (dissection, thrombophilia, paraneoplastic, etc.) and unknown causes, might, however, be inconclusive due to a possible lack of standardized classification of etiology across studies (49).

It is thought that NIHSS gives more weight to neurological deficits in anterior circulation stroke due to factors such as aphasia, facial palsy, and hemiparesis as opposed to limb ataxia, oculomotor disorders, and hemianopia in posterior circulation stroke. However, depending on the level of occlusion, some cases of PCLVO are accompanied by hemiparesis, facial palsy, and dysarthria. It could therefore be argued that a substantial overlap in clinical characteristics exists in both anterior and posterior circulation strokes (14, 50). Contrary to the presumption that NIHSS gives more weight to neurological deficits in anterior circulation strokes, our study detected a higher admission NIHSS in PCLVO than in ACLVO (Figure 4). This could be due to more PCLVO patients with reduced consciousness on admission although we do not have data to support this presumption.

Our meta-analysis showed that fewer PCLVO patients are likely to receive IVT in comparison to ACLVO (Figure 5). Previous literature have reported prodromal symptoms in up to 60% cases of PCLVO, which, in most cases, is a reason for misdiagnosis and wrong specialty consultation (17, 51). As a result, PCLVO patients may not succeed presenting within the widely accepted 4.5-h time window to receive IVT (52).

Furthermore, the delay in neurological intervention in patients with posterior circulation stroke was reflected in the longer onset-to-IVT and onset-to-groin puncture times in PCLVO (Figures 6, 7). This association is supported by previous studies (53). In spite of the longer onset-to-IVT and onset-to-groin puncture times in PCLVO, a favorable 90 day functional outcome in PCLVO is, however, equally possible just as in ACLVO (Figure 10). This could support the hypothesis that salvageable brain tissue in posterior circulation stroke persists for a longer time as compared to anterior circulation stroke possibly due to a better collateralization in the brainstem (54). Shorter onset-to-IVT and onset-to-groin puncture times could therefore influence a better functional MT outcome in PCLVO. Although onset-to-recanalization time and number of passages were comparable between PCLVO and ACLVO, they tended to be increased in PCLVO patients (Supplementary Figures 7, 8). MT in PCLVO was, however, shown to be associated with longer onset-to-recanalization times in the sensitivity analysis, which was not surprising due to the known delays in hospital admission and intervention of PCLVO patients (14, 53).

In our study, we found lower likelihood of sICH in PCLVO (Figure 9). Previous literature has attributed the scarcity of sICH in posterior circulation stroke to relatively smaller infarct volumes and the anatomically smaller nature of vessels that supply the brainstem and cerebellum (55, 56). However, this could also be attributed to the lower number of PCLVO patients who receive IVT (Figure 5). Several other studies have shown IVT in posterior circulation stroke to be associated with lower occurrence of sICH than IVT in anterior circulation stroke (57, 58). Similar results have been demonstrated by a more recent meta-analysis that indicated a lower likelihood of sICH after IVT in posterior circulation stroke (59). On the other hand, a randomized clinical trial that enrolled 656 patients showed no significant difference in sICH in IVT and non-IVT patients although this study included patients with either ACLVO or PCLVO (60). This raises the question as to whether sICH after MT in PCLVO could therefore be independent of IVT administration. In the anterior circulation, however, MT in addition to IVT has been identified as a significant independent predictor of ICH (56, 61).

Studies by previous meta-analyses showed no statistical difference between PCLVO and ACLVO in both recanalization success and 90 day functional outcome (19, 20). A comparable likelihood of obtaining successful recanalization (Figure 8) and 90 day favorable functional outcome (Figure 10) was also found in our meta-analysis that included more recent studies. A successful recanalization could therefore influence a good functional outcome irrespective of the circulation involved. Although this study makes a comparison between MT in PCLVO vs. ACLVO, we believe that RCTs are warranted to study if MT in PCLVO is generally efficient.

The incidence of higher mortality in PCLVO has been discussed in numerous previous studies (19, 20). Our study likewise provided data to support the claim that MT in PCLVO is associated with a higher mortality as compared to ACLVO (Figure 11). Although it is believed that younger patients tend to have a better stroke outcome in comparison to older patients (55, 56), this study shows that PCLVO patients are younger but yet are still associated with a higher mortality than ACLVO patients.

Futile recanalization, a phenomenon defined as poor functional outcome with mRS 4–6, despite successful recanalization by MT, have been reported in individual studies as being significantly higher in PCLVO than ACLVO [OR = 2.15 (95% CI 1.27–3.63)] (39).

Due to the higher probability of futile recanalization, physicians may be more conservative and may not attempt MT in older PCLVO patients, hence creating a selection bias with higher numbers of younger patients being considered for MT. It is, however, worth mentioning that this study does not include data on futile recanalization and physicians' patient management.

The higher rate of mortality in PCLVO compared to ACLVO could be partly due to the relatively higher NIHSS on admission and, hence, stroke severity in posterior circulation stroke (Figure 4). This is in line with previous suggestions that stroke severity on admission is an important predictor of stroke outcome, especially in the posterior circulation, and that higher baseline NIHSS in PCLVO is associated with a poor outcome (24, 62).

In addition, stroke due to basilar artery occlusion has been described as severe in relation to other occlusion sites in PCLVO (63). We reported basilar artery occlusion as the most frequent site of PCLVO (33.5%) (Table 1), which may also have contributed to the higher mortality. This study therefore suggests onset-to-IVT and onset-to-groin puncture times, NIHSS, and basilar artery occlusion as factors that could influence outcome in PCLVO. As a reason for high mortality in PCLVO, we propose a subtle progressive-over-time damage or a non-life supporting damage in the posterior circulation, possibly due to the gravity and irreversible nature of the damage to support life despite neurorehabilitation. Such damages have been described as a comatose state or locked-in syndrome, dysphagia, tracheostomy, hypostatic pneumonia, and complications as a result of being long-term bedridden (42). However, this hypothesis cannot be supported by the present study and we therefore encourage further studies to detect causes of higher mortality of MT in PCLVO.

### Limitations

Although we implemented measures to limit setbacks in this study, we were nonetheless posed with a couple of challenges. Firstly, such a meta-analysis with several studies over such long duration faces the problem of high heterogeneity with respect to stroke management across studies. Secondly, although efforts were made to exclude the possible effects of the use of first-generation MT devices, there was no 100% guarantee that all remaining studies included in our subgroup analyses exclusively used second-generation MT devices. Thirdly, there was a huge disparity in number of PCLVO and ACLVO patients. Finally, the lack of RCT in both groups introduces selection bias.

## Conclusion

Although MT in PCLVO differs characteristically and also in terms of outcome from ACLVO, our meta-analysis indicates that MT in PCLVO may be equally efficient just as in ACLVO in achieving successful recanalization and a favorable 90 day functional outcome. Although MT in PCLVO is associated with lower likelihood of sICH, possibly due to fewer PCLVO patients receiving IVT because of late recognition and presentation, PCLVO is associated with a higher occurrence of mortality. This higher mortality could be explained through the high baseline NIHSS, longer onset-to-IVT and onset-to-groin puncture times, and basilar artery occlusion being the most predominant site of PCLVO.

## Data Availability Statement

The original contributions presented in the study are included in the article/Supplementary Material, further inquiries can be directed to the corresponding author/s.

## Author Contributions

JM and SP conceived and designed the study, undertook data extraction, analyzed the data, and drafted the manuscript. KP and JT independently rechecked all extracted data and analysis. AG-E, YW, KF, and AM helped with data analysis. UZ helped interpret the data. SP had full access to all the data in the study and had final responsibility for the decision to submit for publication. All authors reviewed and edited the manuscript and approved the final version of the manuscript.

## Conflict of Interest

UZ received research grants from BMS, European Research Council, German Federal Ministry of Education and Research, German Research Foundation, Janssen Pharmaceuticals, and Takeda, and personal consulting fees from Bayer, CorTec, and Pfizer. SP received research grants from BMS/Pfizer, Daiichi Sankyo, European Union, German Federal Joint Committee Innovation Fund, and German Federal Ministry of Education and Research, and speakers' honoraria/consulting fees from AstraZeneca, Bayer, Boehringer-Ingelheim, BMS/Pfizer, Daiichi Sankyo, Portola, and Werfen. The remaining authors declare that the research was conducted in the absence of any commercial or financial relationships that could be construed as a potential conflict of interest.
