# Supplementary material for: Comparison of Risk Factors, Safety, and Efficacy Outcomes of Mechanical Thrombectomy in Posterior vs. Anterior Circulation Large Vessel Occlusion
Source: Front Neurol. 2021 Jun 22;12:687134. doi: 10.3389/fneur.2021.687134 (PMC8258169; doi:10.3389/fneur.2021.687134)
Supplement: Supplementary file 1 [file Data_Sheet_1.pdf]

Supplementary Material

Supplementary Methods

Search string for meta-analysis

("thrombectomy"[MeSH Terms] OR "thrombectomy"[All Fields] OR "thrombectomies"[All Fields] OR "endovascular"[All Fields] OR ("intervention s"[All Fields] OR "interventions"[All Fields] OR "interventive"[All Fields] OR "methods"[MeSH Terms] OR "methods"[All Fields] OR "intervention"[All Fields] OR "interventional"[All Fields])) AND ((("posterior"[All Fields] OR "posteriors"[All Fields]) AND ("blood circulation"[MeSH Terms] OR ("blood"[All Fields] AND "circulation"[All Fields]) OR "blood circulation"[All Fields] OR "circulation"[All Fields] OR "circulations"[All Fields] OR "circulate"[All Fields] OR "circulated"[All Fields] OR "circulates"[All Fields] OR "circulating"[All Fields])) OR "basilar"[All Fields]) AND (("anterior"[All Fields] OR "anteriores"[All Fields] OR "anteriorization"[All Fields] OR "anteriorized"[All Fields] OR "anteriors"[All Fields]) AND ("blood circulation"[MeSH Terms] OR ("blood"[All Fields] AND "circulation"[All Fields]) OR "blood circulation"[All Fields] OR "circulation"[All Fields] OR "circulations"[All Fields] OR "circulate"[All Fields] OR "circulated"[All Fields] OR "circulates"[All Fields] OR "circulating"[All Fields])) AND ("stroke"[MeSH Terms] OR "stroke"[All Fields] OR "strokes"[All Fields] OR "stroke s"[All Fields])

Supplementary Figures

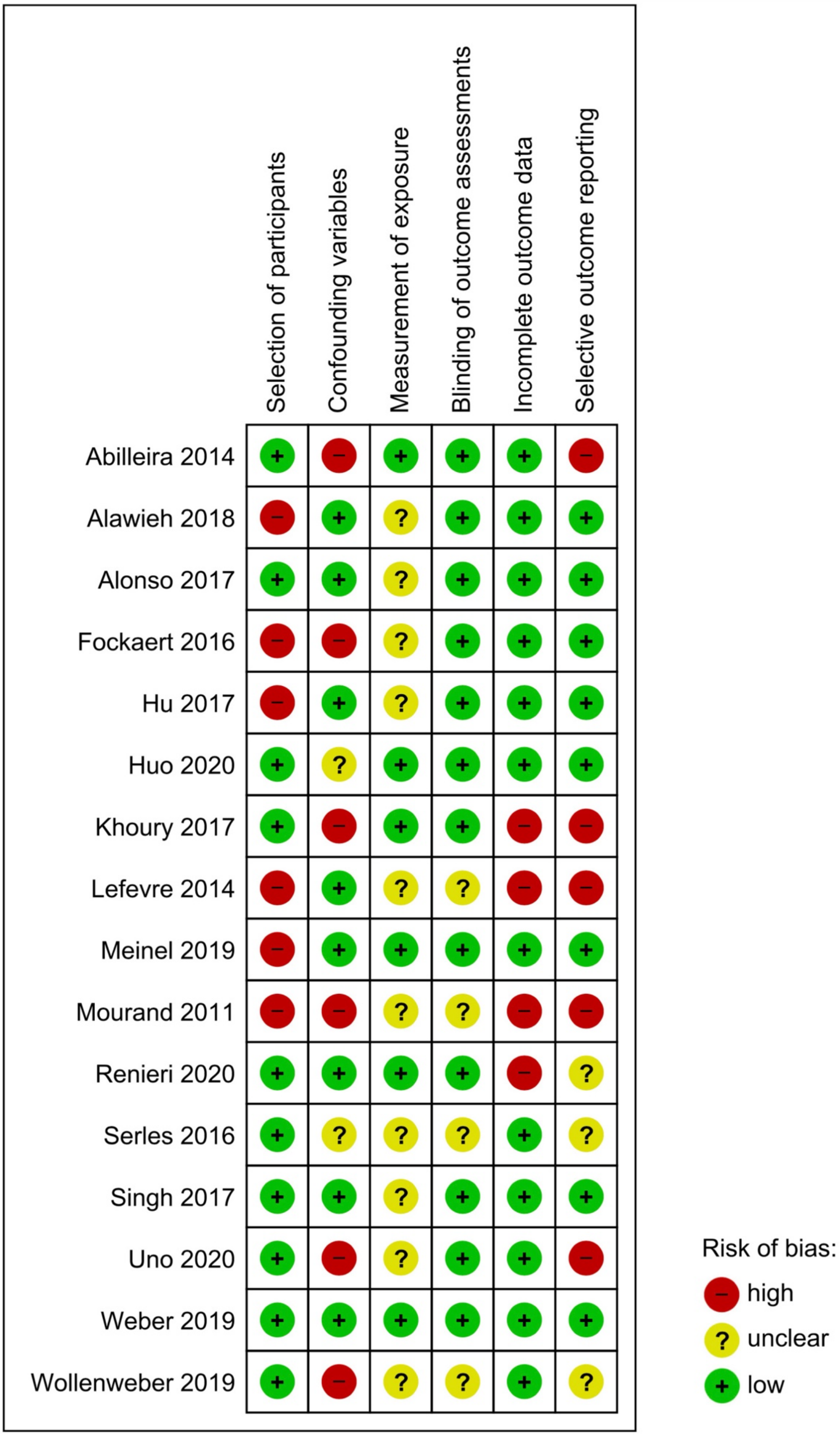

Supplementary Figure 1: Risk of Bias Assessment tool for Non-randomized Studies (RoBANS) for included studies (Kim et al., 2013)

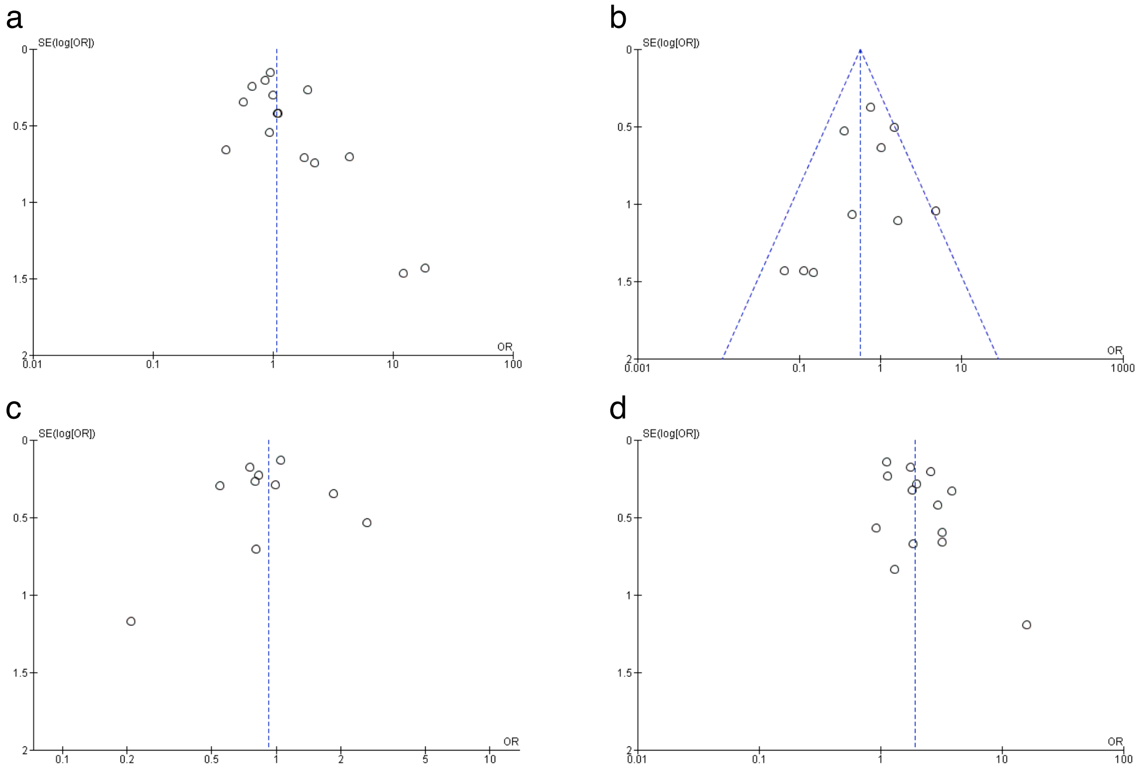

Supplementary Figure 2: Funnel plots for detecting asymmetry and publication bias of (a) successful recanalization defined as thrombolysis in cerebral infarction scale (TICI) 2/3, (b) symptomatic intracranial hemorrhage, (c) favorable functional outcome defined as modified Rankin Scale score 0–2 at 90 days, (d) mortality at 90 days

A

Study or Subgroup	PC Events	PC Total	AC Events	AC Total	Weight	Odds Ratio M-H, Random, 95% CI	Year
Abilleira 2014	37	65	284	471	11.7%	0.87 [0.51, 1.47]	2014
Alonso 2017	27	52	268	427	11.1%	0.64 [0.36, 1.14]	2017
Hu 2017	8	24	58	136	7.7%	0.67 [0.27, 1.68]	2017
Singh 2017	11	25	56	112	8.0%	0.79 [0.33, 1.88]	2017
Alawieh 2018	15	56	148	380	10.5%	0.57 [0.31, 1.07]	2018
Meinel 2019	87	163	1046	1553	13.8%	0.55 [0.40, 0.77]	2019
Weber 2019	94	139	745	961	13.2%	0.61 [0.41, 0.89]	2019
Huo 2020	98	145	298	596	13.2%	2.09 [1.42, 3.06]	2020
Uno 2020	26	50	184	295	10.8%	0.65 [0.36, 1.19]	2020
**Total (95% CI)**		**719**		**4931**	**100.0%**	**0.76 [0.54, 1.09]**	
Total events	403		3087				

Heterogeneity: Tau² = 0.21; Chi² = 33.20, df = 8 (P < 0.0001); I² = 76%
Test for overall effect: Z = 1.49 (P = 0.14)

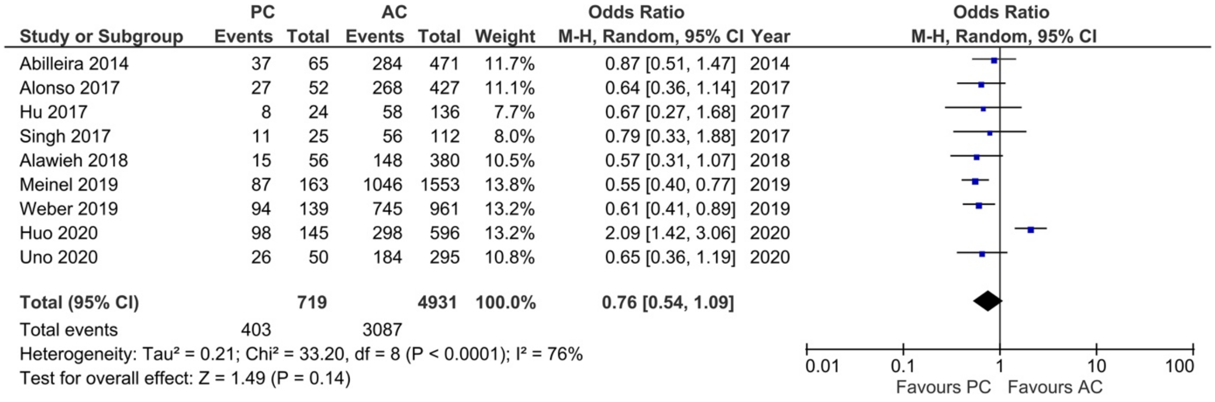

B

Study or Subgroup	PC Events	PC Total	AC Events	AC Total	Weight	Odds Ratio M-H, Random, 95% CI	Year	A	B	C	D	E	F
Abilleira 2014	37	65	284	471	17.1%	0.87 [0.51, 1.47]	2014	+	−	+	+	+	−
Alonso 2017	27	52	268	427	16.4%	0.64 [0.36, 1.14]	2017	+	+	?	+	+	+
Hu 2017	8	24	58	136	0.0%	0.67 [0.27, 1.68]	2017	−	+	?	+	+	+
Singh 2017	11	25	56	112	12.5%	0.79 [0.33, 1.88]	2017	+	+	?	+	+	+
Alawieh 2018	15	56	148	380	0.0%	0.57 [0.31, 1.07]	2018	−	+	?	+	+	+
Meinel 2019	87	163	1046	1553	0.0%	0.55 [0.40, 0.77]	2019	−	+	+	+	+	+
Weber 2019	94	139	745	961	18.9%	0.61 [0.41, 0.89]	2019	+	+	+	+	+	+
Huo 2020	98	145	298	596	19.0%	2.09 [1.42, 3.06]	2020	+	?	+	+	+	+
Uno 2020	26	50	184	295	16.1%	0.65 [0.36, 1.19]	2020	+	−	?	+	+	−
**Total (95% CI)**		**476**		**2862**	**100.0%**	**0.86 [0.53, 1.38]**							
Total events	293		1835										

Heterogeneity: Tau² = 0.27; Chi² = 24.98, df = 5 (P = 0.0001); I² = 80%
Test for overall effect: Z = 0.62 (P = 0.53)

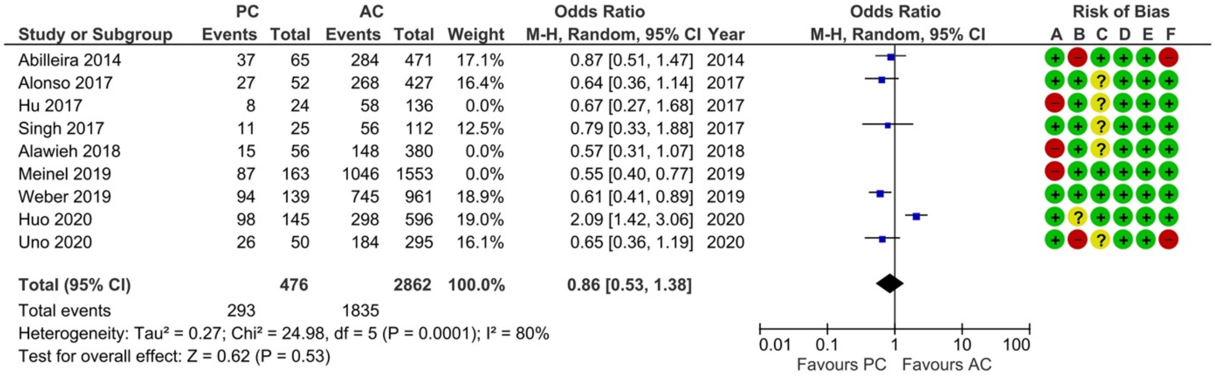

Risk of bias legend
(**A**) Selection of participants
(**B**) Confounding variables
(**C**) Measurement of exposure
(**D**) Blinding of outcome assessments
(**E**) Incomplete outcome data
(**F**) Selective outcome reporting

Supplementary Figure 3: Forest plot comparing comorbidity 'hypertension' of patients with large vessel occlusion in the posterior circulation (PC) versus anterior circulation (AC) who were treated with endovascular mechanical thrombectomy; A: results for all studies, B: results for sensitivity analysis which excluded studies on the basis of high risk of selection bias.

Chi^2 = chi-square statistic, CI = confidence interval, df = degrees of freedom, I^2 = I-square heterogeneity statistic, M-H = Mantel-Haenszel statistic, P = p value, Tau^2 = estimated variance of underlying effects across studies, Z = Z statistic

A

Study or Subgroup	PC Events	PC Total	AC Events	AC Total	Weight	Odds Ratio M-H, Fixed, 95% CI	Year
Abilleira 2014	13	65	145	471	12.1%	0.56 [0.30, 1.06]	2014
Fockaert 2016	4	15	29	65	3.4%	0.45 [0.13, 1.57]	2016
Alonso 2017	13	52	175	427	12.2%	0.48 [0.25, 0.93]	2017
Hu 2017	13	24	84	136	5.0%	0.73 [0.31, 1.75]	2017
Singh 2017	1	25	5	112	0.8%	0.89 [0.10, 7.98]	2017
Alawieh 2018	40	56	261	380	8.2%	1.14 [0.61, 2.12]	2018
Weber 2019	42	139	411	961	31.1%	0.58 [0.39, 0.85]	2019
Huo 2020	20	145	135	596	19.6%	0.55 [0.33, 0.91]	2020
Uno 2020	10	50	76	295	7.6%	0.72 [0.34, 1.51]	2020
**Total (95% CI)**		**571**		**3443**	**100.0%**	**0.62 [0.50, 0.77]**	
Total events	156		1321				

Heterogeneity: Chi² = 5.40, df = 8 (P = 0.71); I² = 0%
Test for overall effect: Z = 4.44 (P < 0.00001)

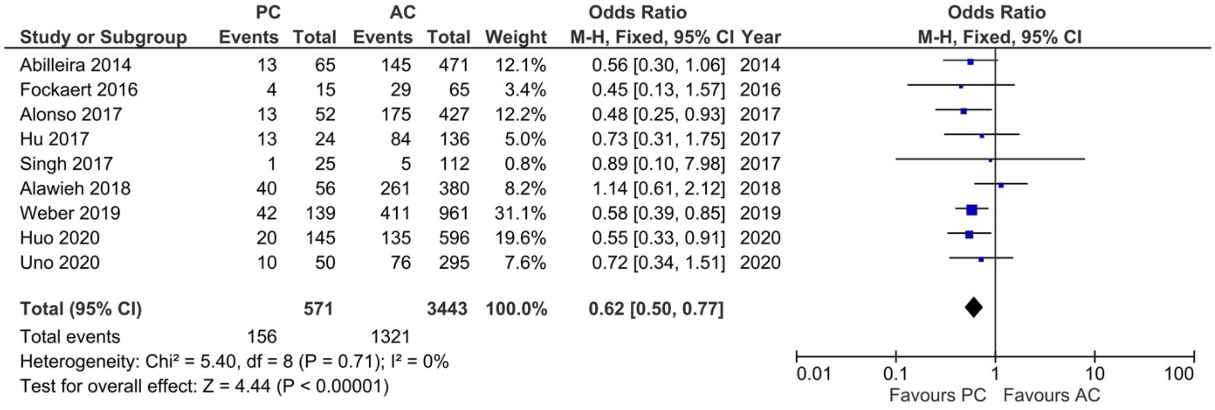

B

Study or Subgroup	PC Events	PC Total	AC Events	AC Total	Weight	Odds Ratio M-H, Fixed, 95% CI	Year	A	B	C	D	E	F
Abilleira 2014	13	65	145	471	14.5%	0.56 [0.30, 1.06]	2014	+	−	+	+	+	−
Fockaert 2016	4	15	29	65	0.0%	0.45 [0.13, 1.57]	2016	−	−	?	+	+	+
Alonso 2017	13	52	175	427	14.7%	0.48 [0.25, 0.93]	2017	+	+	?	+	+	+
Hu 2017	13	24	84	136	0.0%	0.73 [0.31, 1.75]	2017	−	+	?	+	+	+
Singh 2017	1	25	5	112	0.9%	0.89 [0.10, 7.98]	2017	+	+	?	+	+	+
Alawieh 2018	40	56	261	380	0.0%	1.14 [0.61, 2.12]	2018	−	+	?	+	+	+
Weber 2019	42	139	411	961	37.4%	0.58 [0.39, 0.85]	2019	+	+	+	+	+	+
Huo 2020	20	145	135	596	23.5%	0.55 [0.33, 0.91]	2020	+	?	+	+	+	+
Uno 2020	10	50	76	295	9.1%	0.72 [0.34, 1.51]	2020	+	−	?	+	+	−
**Total (95% CI)**		**476**		**2862**	**100.0%**	**0.57 [0.45, 0.72]**							
Total events	99		947										

Heterogeneity: Chi² = 0.84, df = 5 (P = 0.97); I² = 0%
Test for overall effect: Z = 4.60 (P < 0.00001)

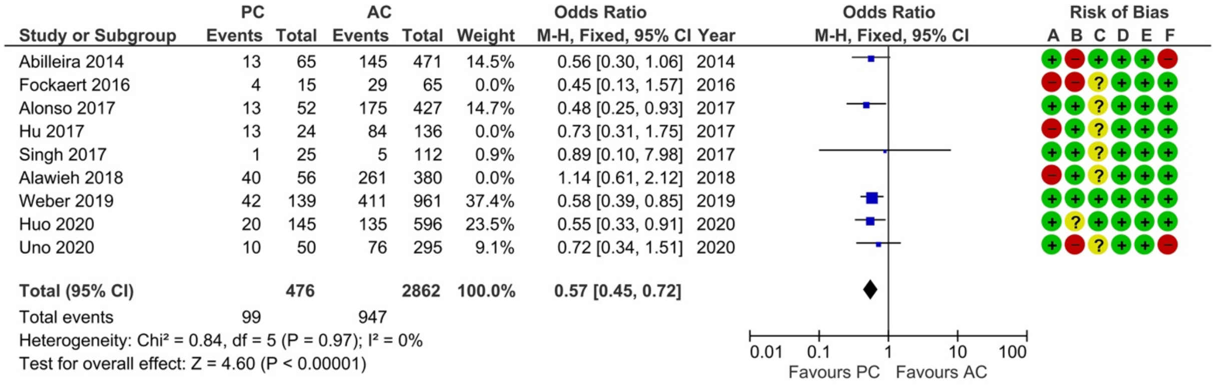

Risk of bias legend
(**A**) Selection of participants
(**B**) Confounding variables
(**C**) Measurement of exposure
(**D**) Blinding of outcome assessments
(**E**) Incomplete outcome data
(**F**) Selective outcome reporting

Supplementary Figure 4: Forest plot comparing comorbidity 'atrial fibrillation' of patients with large vessel occlusion in the posterior circulation (PC) versus anterior circulation (AC) who were treated with endovascular mechanical thrombectomy; A: results for all studies, B: results for sensitivity analysis which excluded studies on the basis of high risk of selection bias.

Chi^2 = chi-square statistic, CI = confidence interval, df = degrees of freedom, I^2 = I-square heterogeneity statistic, M-H = Mantel-Haenszel statistic, P = p value, Z = Z statistic

A

Study or Subgroup	PC Events	PC Total	AC Events	AC Total	Weight	Odds Ratio M-H, Fixed, 95% CI	Year
Abilleira 2014	22	65	169	471	10.5%	0.91 [0.53, 1.58]	2014
Alonso 2017	20	52	193	427	10.0%	0.76 [0.42, 1.37]	2017
Hu 2017	7	24	37	136	3.1%	1.10 [0.42, 2.87]	2017
Alawieh 2018	15	56	152	380	11.1%	0.55 [0.29, 1.03]	2018
Weber 2019	44	139	346	961	23.2%	0.82 [0.56, 1.20]	2019
Meinel 2019	60	161	788	1547	36.2%	0.57 [0.41, 0.80]	2019
Uno 2020	14	50	73	295	5.9%	1.18 [0.60, 2.31]	2020
**Total (95% CI)**		**547**		**4217**	**100.0%**	**0.73 [0.61, 0.89]**	
Total events	182		1758				

Heterogeneity: Chi² = 6.56, df = 6 (P = 0.36); I² = 9%

Test for overall effect: Z = 3.19 (P = 0.001)

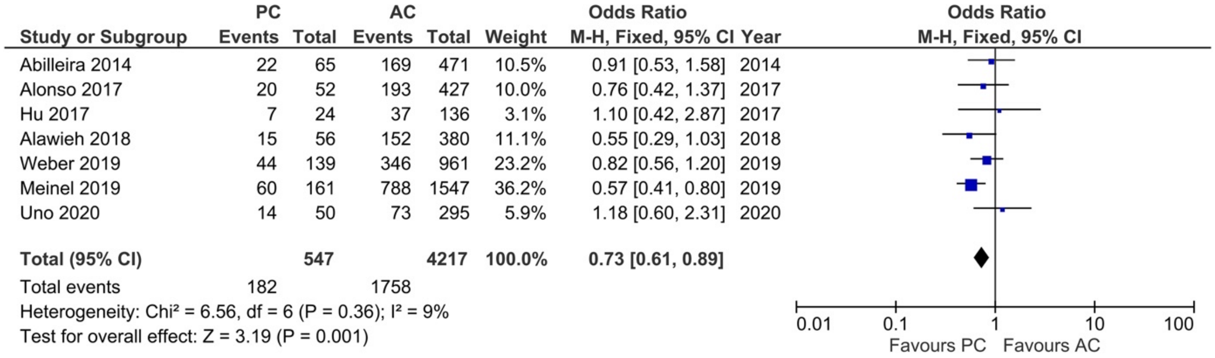

B

Study or Subgroup	PC Events	PC Total	AC Events	AC Total	Weight	Odds Ratio M-H, Fixed, 95% CI	Year	Risk of Bias A	B	C	D	E	F
Abilleira 2014	22	65	169	471	21.2%	0.91 [0.53, 1.58]	2014	+	−	+	+	+	−
Alonso 2017	20	52	193	427	20.2%	0.76 [0.42, 1.37]	2017	+	+	?	+	+	+
Hu 2017	7	24	37	136	0.0%	1.10 [0.42, 2.87]	2017	−	+	?	+	+	+
Alawieh 2018	15	56	152	380	0.0%	0.55 [0.29, 1.03]	2018	−	+	?	+	+	+
Weber 2019	44	139	346	961	46.7%	0.82 [0.56, 1.20]	2019	+	+	+	+	+	+
Meinel 2019	60	161	788	1547	0.0%	0.57 [0.41, 0.80]	2019	−	+	+	+	+	+
Uno 2020	14	50	73	295	11.9%	1.18 [0.60, 2.31]	2020	+	−	?	+	+	−
**Total (95% CI)**		**306**		**2154**	**100.0%**	**0.87 [0.68, 1.13]**							
Total events	100		781										

Heterogeneity: Chi² = 1.12, df = 3 (P = 0.77); I² = 0%

Test for overall effect: Z = 1.05 (P = 0.29)

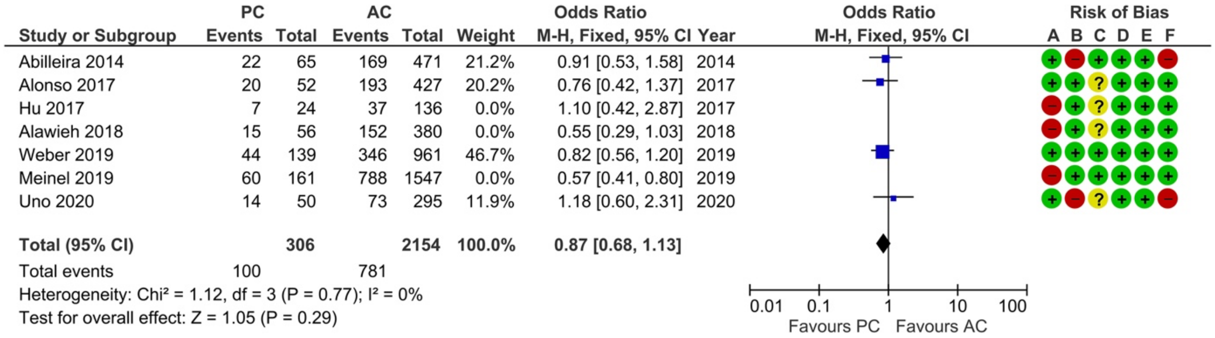

Risk of bias legend

(**A**) Selection of participants
(**B**) Confounding variables
(**C**) Measurement of exposure
(**D**) Blinding of outcome assessments
(**E**) Incomplete outcome data
(**F**) Selective outcome reporting

Supplementary Figure 5: Forest plot comparing comorbidity 'hyperlipidemia' of patients with large vessel occlusion in the posterior circulation (PC) versus anterior circulation (AC) who were treated with endovascular mechanical thrombectomy; A: results for all studies, B: results for sensitivity analysis which excluded studies on the basis of high risk of selection bias.

Chi^2 = chi-square statistic, CI = confidence interval, df = degrees of freedom, I^2 = I-square heterogeneity statistic, M-H = Mantel-Haenszel statistic, P = p value, Z = Z statistic

A

Study or Subgroup	PC Events	PC Total	AC Events	AC Total	Weight	Odds Ratio M-H, Fixed, 95% CI	Year
Abilleira 2014	15	65	87	471	8.7%	1.32 [0.71, 2.47]	2014
Singh 2017	19	52	96	427	7.1%	1.99 [1.08, 3.65]	2017
Alonso 2017	6	25	28	112	4.2%	0.95 [0.34, 2.61]	2017
Weber 2019	47	153	428	1499	29.4%	1.11 [0.77, 1.59]	2019
Meinel 2019	30	139	142	961	15.1%	1.59 [1.02, 2.47]	2019
Uno 2020	49	145	195	596	27.1%	1.05 [0.71, 1.54]	2020
Huo 2020	11	50	70	295	8.5%	0.91 [0.44, 1.86]	2020
**Total (95% CI)**		**629**		**4361**	**100.0%**	**1.22 [1.01, 1.48]**	
Total events	177		1046				

Heterogeneity: Chi² = 5.63, df = 6 (P = 0.47); I² = 0%
Test for overall effect: Z = 2.07 (P = 0.04)

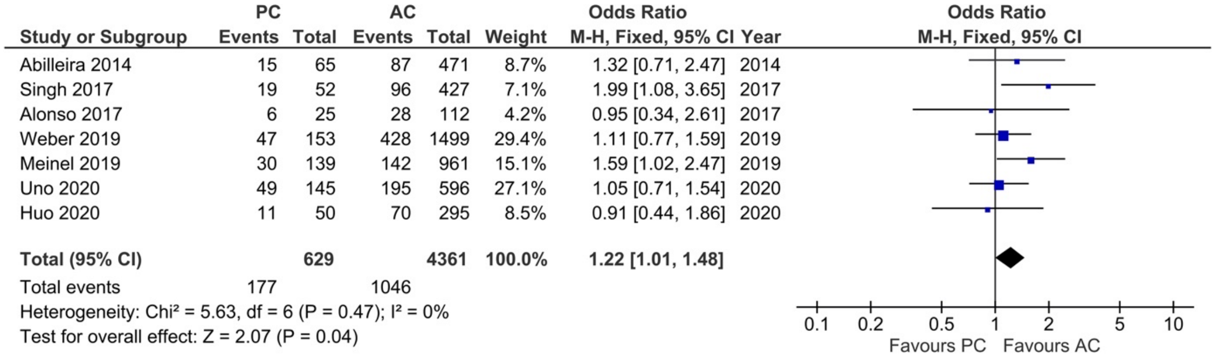

B

Study or Subgroup	PC Events	PC Total	AC Events	AC Total	Weight	Odds Ratio M-H, Fixed, 95% CI	A	B	C	D	E	F
Abilleira 2014	15	65	87	471	10.2%	1.32 [0.71, 2.47]	+	−	+	+	+	−
Alonso 2017	6	25	28	112	4.9%	0.95 [0.34, 2.61]	+	+	?	+	+	+
Huo 2020	11	50	70	295	10.0%	0.91 [0.44, 1.86]	+	?	+	+	+	+
Meinel 2019	30	139	142	961	0.0%	1.59 [1.02, 2.47]	−	+	+	+	+	+
Singh 2017	19	52	96	427	8.3%	1.99 [1.08, 3.65]	+	+	?	+	+	+
Uno 2020	49	145	195	596	31.9%	1.05 [0.71, 1.54]	+	−	?	+	+	−
Weber 2019	47	153	428	1499	34.7%	1.11 [0.77, 1.59]	+	+	+	+	+	+
**Total (95% CI)**		**490**		**3400**	**100.0%**	**1.16 [0.94, 1.43]**						
Total events	147		904									

Heterogeneity: Chi² = 4.09, df = 5 (P = 0.54); I² = 0%
Test for overall effect: Z = 1.36 (P = 0.17)

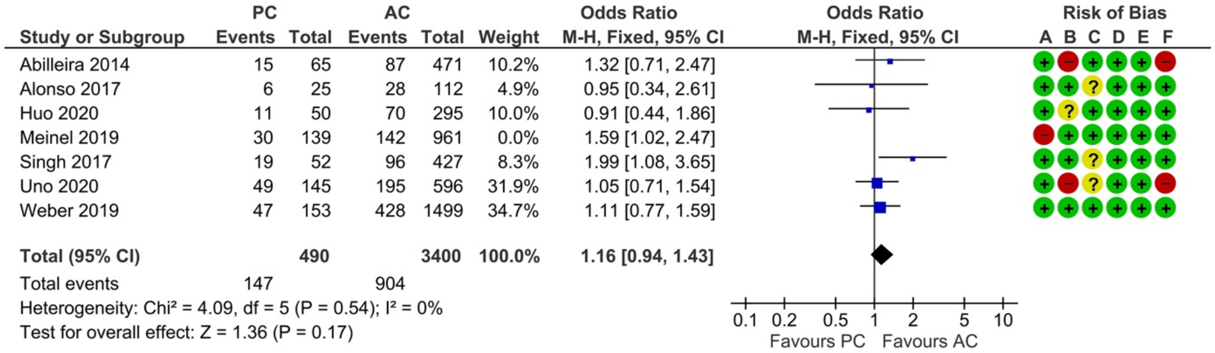

Risk of bias legend
(**A**) Selection of participants
(**B**) Confounding variables
(**C**) Measurement of exposure
(**D**) Blinding of outcome assessments
(**E**) Incomplete outcome data
(**F**) Selective outcome reporting

Supplementary Figure 6: Forest plot comparing comorbidity 'smoking' of patients with large vessel occlusion in the posterior circulation (PC) versus anterior circulation (AC) who were treated with endovascular mechanical thrombectomy; A: results for all studies, B: results for sensitivity analysis which excluded studies on the basis of high risk of selection bias.

Chi^2 = chi-square statistic, CI = confidence interval, df = degrees of freedom, I^2 = I-square heterogeneity statistic, M-H = Mantel-Haenszel statistic, P = p value, Z = Z statistic

A

Study or Subgroup	PC Events	PC Total	AC Events	AC Total	Weight	Odds Ratio M-H, Random, 95% CI	Year
Abilleira 2014	17	65	93	471	12.3%	1.44 [0.79, 2.62]	2014
Alonso 2017	8	52	81	427	9.1%	0.78 [0.35, 1.71]	2017
Hu 2017	9	24	42	136	7.7%	1.34 [0.54, 3.31]	2017
Singh 2017	11	25	24	112	7.7%	2.88 [1.16, 7.15]	2017
Alawieh 2018	11	56	110	380	10.6%	0.60 [0.30, 1.20]	2018
Meinel 2019	23	163	271	1555	15.2%	0.78 [0.49, 1.23]	2019
Weber 2019	19	139	191	961	14.1%	0.64 [0.38, 1.06]	2019
Huo 2020	27	145	85	596	14.8%	1.38 [0.85, 2.22]	2020
Uno 2020	7	50	59	295	8.4%	0.65 [0.28, 1.52]	2020
**Total (95% CI)**		**719**		**4933**	**100.0%**	**0.98 [0.72, 1.34]**	
Total events	132		956				

Heterogeneity: Tau² = 0.11; Chi² = 16.27, df = 8 (P = 0.04); I² = 51%
Test for overall effect: Z = 0.12 (P = 0.91)

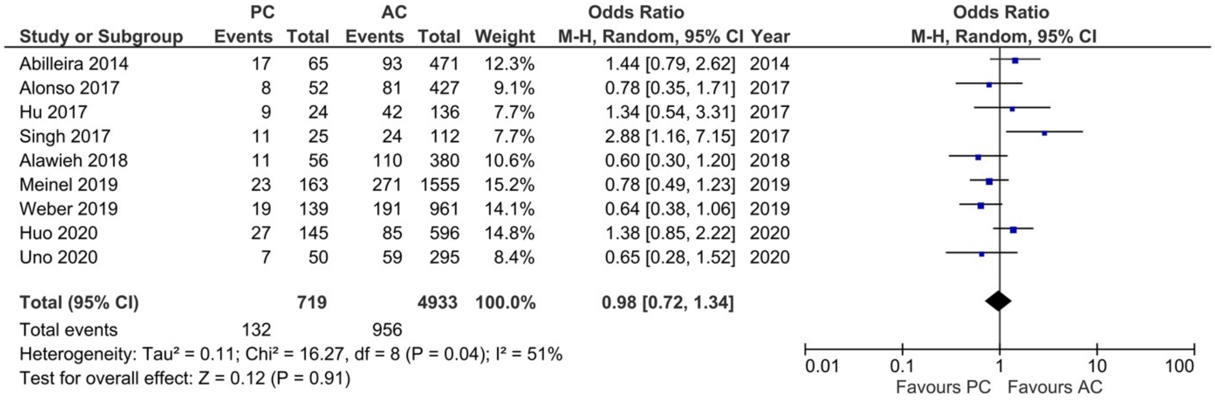

B

Study or Subgroup	PC Events	PC Total	AC Events	AC Total	Weight	Odds Ratio M-H, Random, 95% CI	Year	A	B	C	D	E	F
Abilleira 2014	17	65	93	471	18.3%	1.44 [0.79, 2.62]	2014	+	−	+	+	+	−
Alonso 2017	8	52	81	427	14.4%	0.78 [0.35, 1.71]	2017	+	+	?	+	+	+
Hu 2017	9	24	42	136	0.0%	1.34 [0.54, 3.31]	2017	−	+	?	+	+	+
Singh 2017	11	25	24	112	12.4%	2.88 [1.16, 7.15]	2017	+	+	?	+	+	+
Alawieh 2018	11	56	110	380	0.0%	0.60 [0.30, 1.20]	2018	−	+	?	+	+	+
Meinel 2019	23	163	271	1555	0.0%	0.78 [0.49, 1.23]	2019	−	+	+	+	+	+
Weber 2019	19	139	191	961	20.4%	0.64 [0.38, 1.06]	2019	+	+	+	+	+	+
Huo 2020	27	145	85	596	21.1%	1.38 [0.85, 2.22]	2020	+	?	+	+	+	+
Uno 2020	7	50	59	295	13.4%	0.65 [0.28, 1.52]	2020	+	−	?	+	+	−
**Total (95% CI)**		**476**		**2862**	**100.0%**	**1.08 [0.71, 1.66]**							
Total events	89		533										

Heterogeneity: Tau² = 0.16; Chi² = 12.55, df = 5 (P = 0.03); I² = 60%
Test for overall effect: Z = 0.37 (P = 0.71)

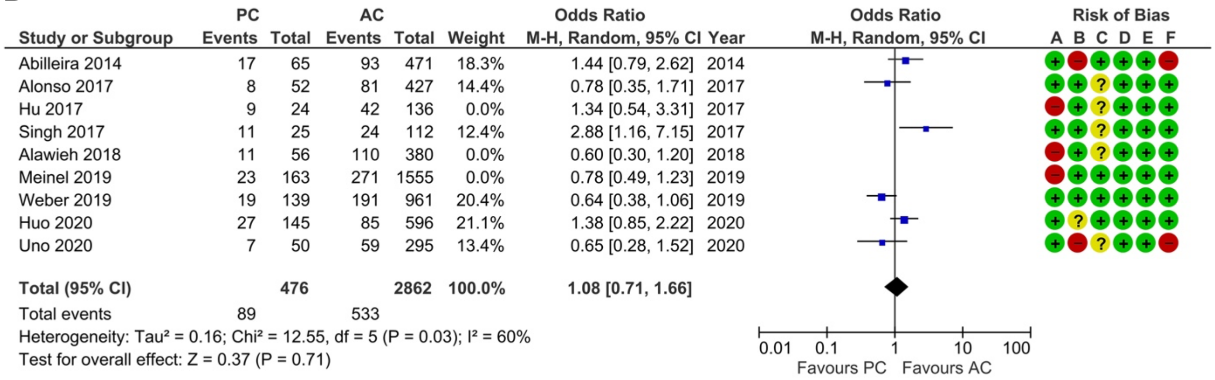

Risk of bias legend
(**A**) Selection of participants
(**B**) Confounding variables
(**C**) Measurement of exposure
(**D**) Blinding of outcome assessments
(**E**) Incomplete outcome data
(**F**) Selective outcome reporting

Supplementary Figure 7: Forest plot comparing comorbidity 'diabetes' of patients with large vessel occlusion in the posterior circulation (PC) versus anterior circulation (AC) who were treated with endovascular mechanical thrombectomy; A: results for all studies, B: results for sensitivity analysis which excluded studies on the basis of high risk of selection bias.

Chi^2 = chi-square statistic, CI = confidence interval, df = degrees of freedom, I^2 = I-square heterogeneity statistic, M-H = Mantel-Haenszel statistic, P = p value, Tau^2 = estimated variance of underlying effects across studies, Z = Z statistic

A

Study or Subgroup	PC Events	PC Total	AC Events	AC Total	Weight	Odds Ratio M-H, Random, 95% CI	Year
Abilleira 2014	13	65	56	471	22.2%	1.85 [0.95, 3.62]	2014
Alonso 2017	4	52	60	427	16.2%	0.51 [0.18, 1.47]	2017
Hu 2017	5	24	65	136	16.4%	0.29 [0.10, 0.81]	2017
Singh 2017	1	25	12	112	7.0%	0.35 [0.04, 2.80]	2017
Meinel 2019	17	83	235	1131	24.0%	0.98 [0.57, 1.71]	2019
Uno 2020	3	50	43	295	14.2%	0.37 [0.11, 1.26]	2020
**Total (95% CI)**		**299**		**2572**	**100.0%**	**0.67 [0.36, 1.27]**	
Total events	43		471				

Heterogeneity: Tau² = 0.36; Chi² = 13.30, df = 5 (P = 0.02); I² = 62%
Test for overall effect: Z = 1.22 (P = 0.22)

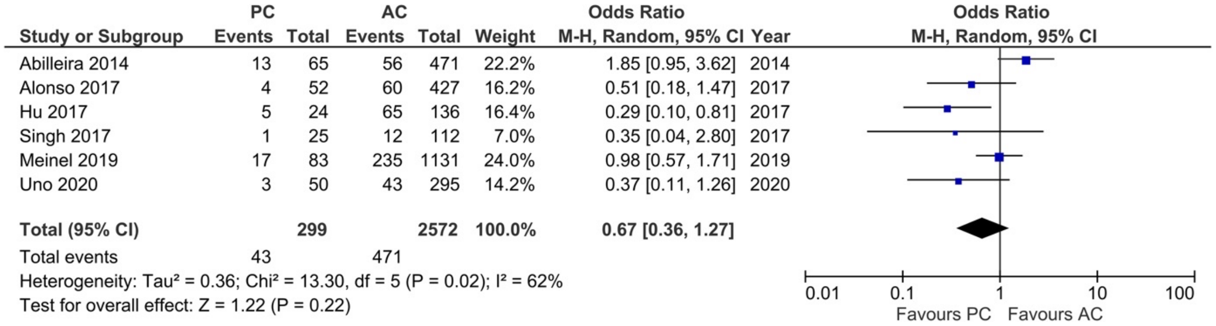

B

Study or Subgroup	PC Events	PC Total	AC Events	AC Total	Weight	Odds Ratio M-H, Random, 95% CI	Year	A	B	C	D	E	F
Abilleira 2014	13	65	56	471	33.9%	1.85 [0.95, 3.62]	2014	+	−	+	+	+	−
Alonso 2017	4	52	60	427	27.3%	0.51 [0.18, 1.47]	2017	+	+	?	+	+	+
Hu 2017	5	24	65	136	0.0%	0.29 [0.10, 0.81]	2017	−	+	?	+	+	+
Singh 2017	1	25	12	112	14.0%	0.35 [0.04, 2.80]	2017	+	+	?	+	+	+
Meinel 2019	17	83	235	1131	0.0%	0.98 [0.57, 1.71]	2019	−	+	+	+	+	+
Uno 2020	3	50	43	295	24.8%	0.37 [0.11, 1.26]	2020	+	−	?	+	+	−
**Total (95% CI)**		**192**		**1305**	**100.0%**	**0.69 [0.26, 1.82]**							
Total events	21		171										

(A–F: Risk of Bias)

Heterogeneity: Tau² = 0.60; Chi² = 8.71, df = 3 (P = 0.03); I² = 66%
Test for overall effect: Z = 0.74 (P = 0.46)

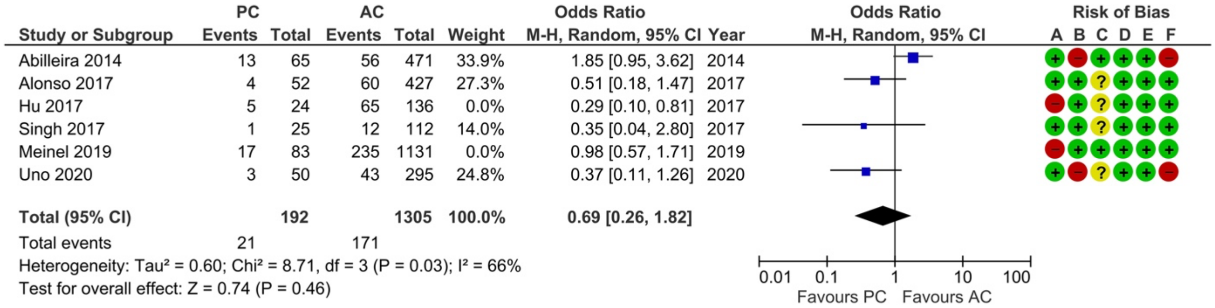

Risk of bias legend
(**A**) Selection of participants
(**B**) Confounding variables
(**C**) Measurement of exposure
(**D**) Blinding of outcome assessments
(**E**) Incomplete outcome data
(**F**) Selective outcome reporting

Supplementary Figure 8: Forest plot comparing comorbidity ‘coronary artery disease’ of patients with large vessel occlusion in the posterior circulation (PC) versus anterior circulation (AC) who were treated with endovascular mechanical thrombectomy; A: results for all studies, B: results for sensitivity analysis which excluded studies on the basis of high risk of selection bias.

Chi^2 = chi-square statistic, CI = confidence interval, df = degrees of freedom, I^2 = I-square heterogeneity statistic, M-H = Mantel-Haenszel statistic, P = p value, Tau^2 = estimated variance of underlying effects across studies, Z = Z statistic

A

Study or Subgroup	PC Events	PC Total	AC Events	AC Total	Weight	Odds Ratio M-H, Fixed, 95% CI	Year
Abilleira 2014	16	65	58	471	8.9%	2.33 [1.24, 4.36]	2014
Alonso 2017	6	52	42	427	6.8%	1.20 [0.48, 2.97]	2017
Hu 2017	1	24	4	136	1.0%	1.43 [0.15, 13.42]	2017
Meinel 2019	17	159	209	1565	28.9%	0.78 [0.46, 1.31]	2019
Weber 2019	26	139	152	961	26.2%	1.22 [0.77, 1.94]	2019
Huo 2020	19	145	58	596	16.5%	1.40 [0.80, 2.43]	2020
Uno 2020	12	50	64	295	11.8%	1.14 [0.56, 2.31]	2020
**Total (95% CI)**		**634**		**4451**	**100.0%**	**1.21 [0.96, 1.53]**	
Total events	97		587				

Heterogeneity: Chi² = 7.22, df = 6 (P = 0.30); I² = 17%
Test for overall effect: Z = 1.60 (P = 0.11)

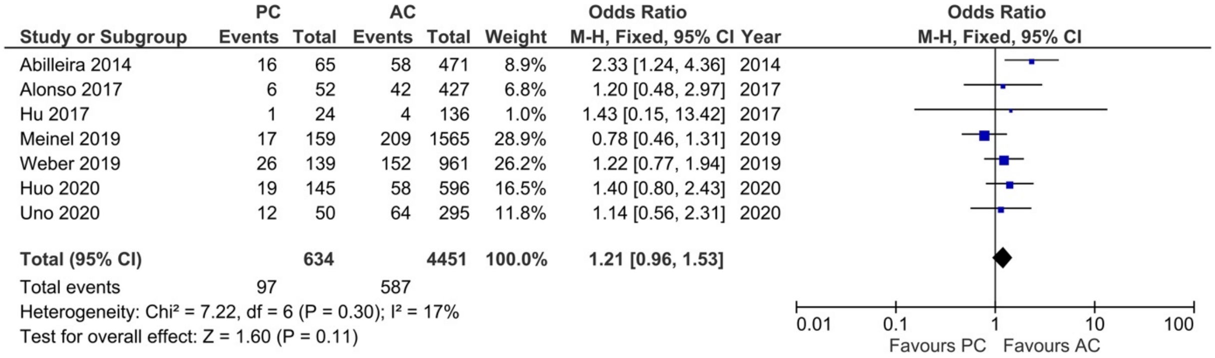

B

Study or Subgroup	PC Events	PC Total	AC Events	AC Total	Weight	Odds Ratio M-H, Fixed, 95% CI	Year	A	B	C	D	E	F
Abilleira 2014	16	65	58	471	12.7%	2.33 [1.24, 4.36]	2014	+	−	+	+	+	−
Alonso 2017	6	52	42	427	9.6%	1.20 [0.48, 2.97]	2017	+	+	?	+	+	+
Hu 2017	1	24	4	136	0.0%	1.43 [0.15, 13.42]	2017	−	+	?	+	+	+
Meinel 2019	17	159	209	1565	0.0%	0.78 [0.46, 1.31]	2019	−	+	+	+	+	+
Weber 2019	26	139	152	961	37.3%	1.22 [0.77, 1.94]	2019	+	+	+	+	+	+
Huo 2020	19	145	58	596	23.6%	1.40 [0.80, 2.43]	2020	+	?	+	+	+	+
Uno 2020	12	50	64	295	16.8%	1.14 [0.56, 2.31]	2020	+	−	?	+	+	−
**Total (95% CI)**		**451**		**2750**	**100.0%**	**1.39 [1.06, 1.82]**							
Total events	79		374										

Heterogeneity: Chi² = 3.28, df = 4 (P = 0.51); I² = 0%
Test for overall effect: Z = 2.39 (P = 0.02)

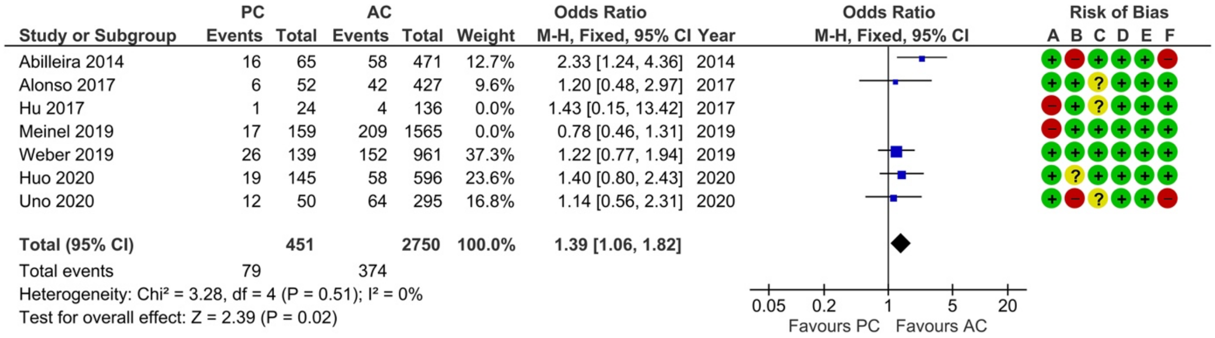

Risk of bias legend
(**A**) Selection of participants
(**B**) Confounding variables
(**C**) Measurement of exposure
(**D**) Blinding of outcome assessments
(**E**) Incomplete outcome data
(**F**) Selective outcome reporting

Supplementary Figure 9: Forest plot comparing comorbidity 'prior stroke or transient ischemic attack' of patients with large vessel occlusion in the posterior circulation (PC) versus anterior circulation (AC) who were treated with endovascular mechanical thrombectomy; A: results for all studies, B: results for sensitivity analysis which excluded studies on the basis of high risk of selection bias.

Chi^2 = chi-square statistic, CI = confidence interval, df = degrees of freedom, I^2 = I-square heterogeneity statistic, M-H = Mantel-Haenszel statistic, P = p value, Z = Z statistic

A

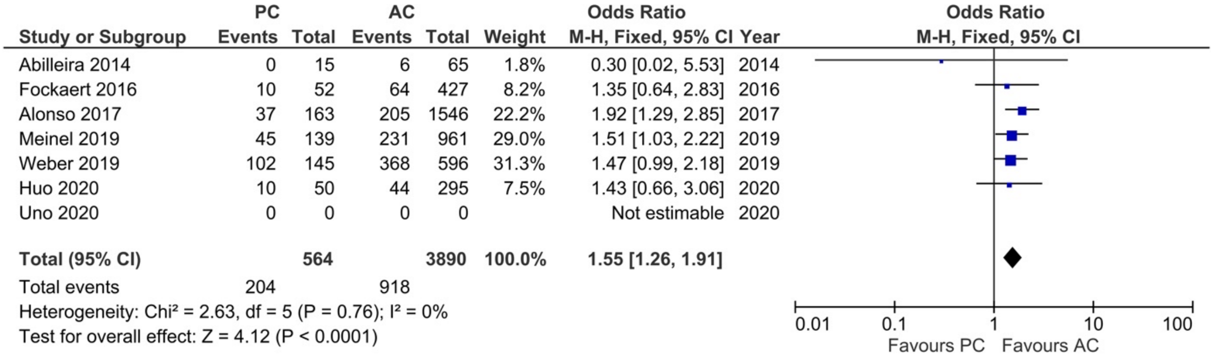

B

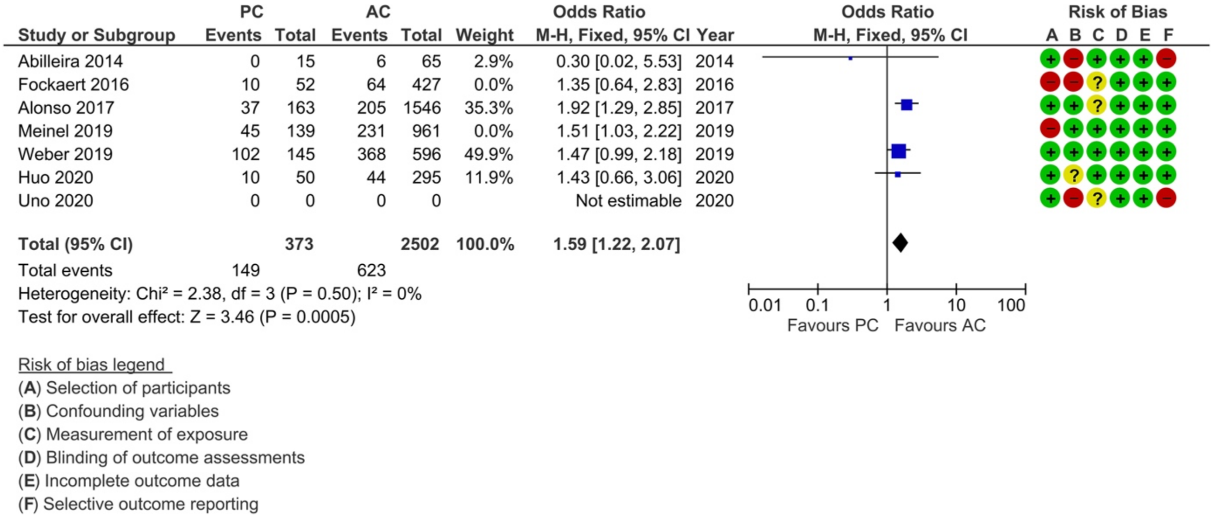

Supplementary Figure 10: Forest plot comparing etiology ‘large artery atherosclerosis’ of patients with large vessel occlusion in the posterior circulation (PC) versus anterior circulation (AC) who were treated with endovascular mechanical thrombectomy; A: results for all studies, B: results for sensitivity analysis which excluded studies on the basis of high risk of selection bias.

Chi^2 = chi-square statistic, CI = confidence interval, df = degrees of freedom, I^2 = I-square heterogeneity statistic, M-H = Mantel-Haenszel statistic, P = p value, Z = Z statistic

A

Study or Subgroup	PC Events	PC Total	AC Events	AC Total	Weight	Odds Ratio M-H, Fixed, 95% CI	Year
Abilleira 2014	23	65	239	471	11.8%	0.53 [0.31, 0.91]	2014
Fockaert 2016	18	43	114	258	6.0%	0.91 [0.47, 1.75]	2016
Serles 2016	6	15	38	65	2.7%	0.47 [0.15, 1.49]	2016
Alonso 2017	18	52	234	427	10.5%	0.44 [0.24, 0.80]	2017
Meinel 2019	64	163	733	1546	26.8%	0.72 [0.52, 1.00]	2019
Weber 2019	46	139	458	961	24.4%	0.54 [0.37, 0.79]	2019
Huo 2020	21	145	121	596	12.8%	0.66 [0.40, 1.10]	2020
Uno 2020	38	50	236	295	5.2%	0.79 [0.39, 1.61]	2020
**Total (95% CI)**		**672**		**4619**	**100.0%**	**0.63 [0.52, 0.75]**	
Total events	234		2173				

Heterogeneity: Chi² = 4.88, df = 7 (P = 0.67); I² = 0%
Test for overall effect: Z = 5.18 (P < 0.00001)

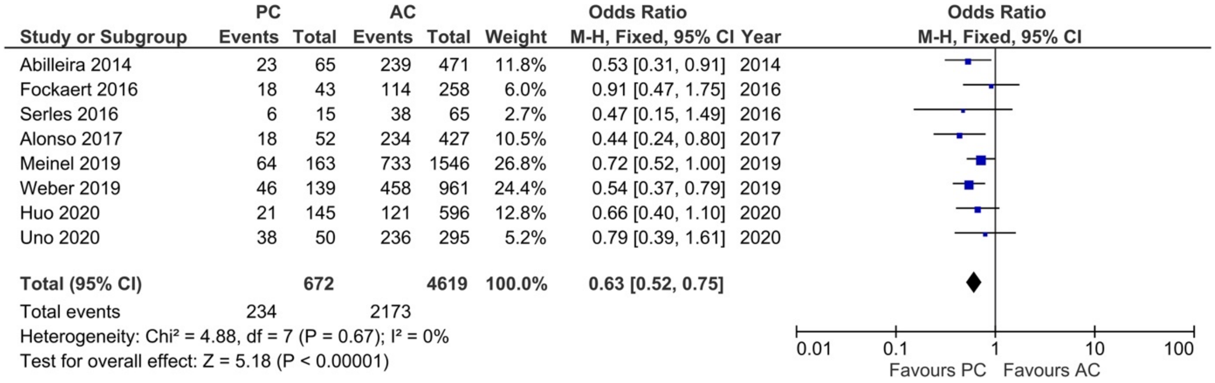

B

Study or Subgroup	PC Events	PC Total	AC Events	AC Total	Weight	Odds Ratio M-H, Fixed, 95% CI	Year	A	B	C	D	E	F
Abilleira 2014	23	65	239	471	17.5%	0.53 [0.31, 0.91]	2014	+	−	+	+	+	−
Serles 2016	6	15	38	65	4.0%	0.47 [0.15, 1.49]	2016	+	?	?	?	+	?
Fockaert 2016	18	43	114	258	0.0%	0.91 [0.47, 1.75]	2016	−	−	?	+	+	+
Alonso 2017	18	52	234	427	15.6%	0.44 [0.24, 0.80]	2017	+	+	?	+	+	+
Meinel 2019	64	163	733	1546	0.0%	0.72 [0.52, 1.00]	2019	−	+	+	+	+	+
Weber 2019	46	139	458	961	36.3%	0.54 [0.37, 0.79]	2019	+	+	+	+	+	+
Huo 2020	21	145	121	596	19.0%	0.66 [0.40, 1.10]	2020	+	?	+	+	+	+
Uno 2020	38	50	236	295	7.7%	0.79 [0.39, 1.61]	2020	+	−	?	+	+	−
**Total (95% CI)**		**466**		**2815**	**100.0%**	**0.56 [0.45, 0.71]**							
Total events	152		1326										

Heterogeneity: Chi² = 2.16, df = 5 (P = 0.83); I² = 0%
Test for overall effect: Z = 5.03 (P < 0.00001)

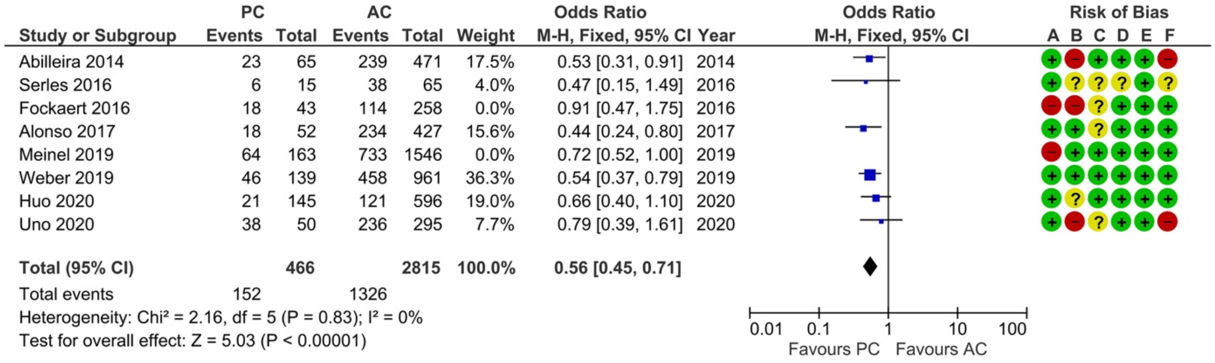

Risk of bias legend
(**A**) Selection of participants
(**B**) Confounding variables
(**C**) Measurement of exposure
(**D**) Blinding of outcome assessments
(**E**) Incomplete outcome data
(**F**) Selective outcome reporting

Supplementary Figure 11: Forest plot comparing etiology 'cardiac embolism' of patients with large vessel occlusion in the posterior circulation (PC) versus anterior circulation (AC) who were treated with endovascular mechanical thrombectomy; A: results for all studies, B: results for sensitivity analysis which excluded studies on the basis of high risk of selection bias.

Chi^2 = chi-square statistic, CI = confidence interval, df = degrees of freedom, I^2 = I-square heterogeneity statistic, M-H = Mantel-Haenszel statistic, P = p value, Z = Z statistic

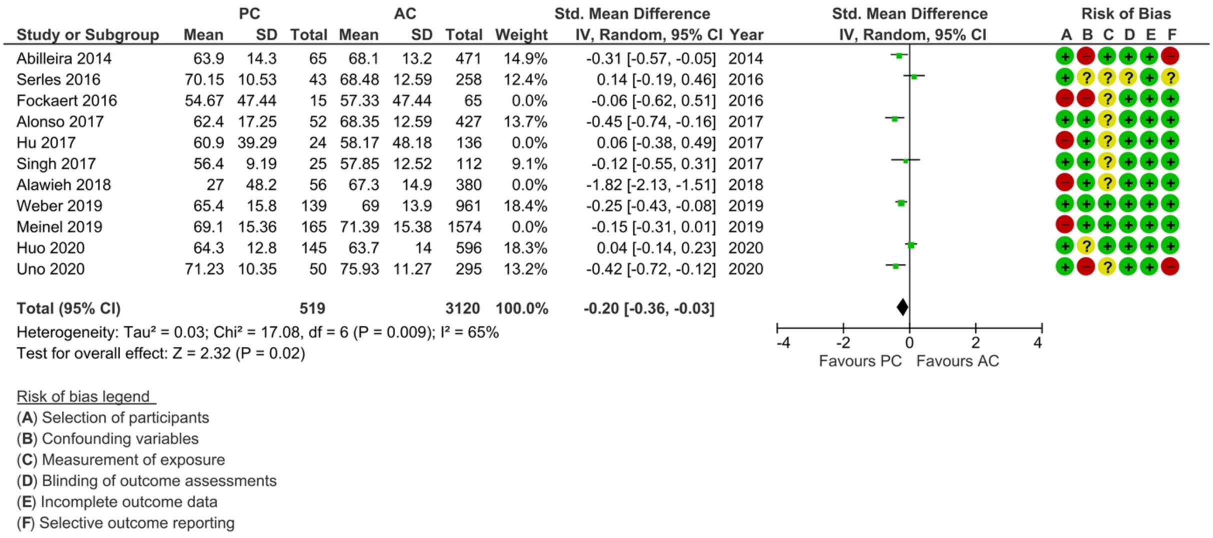

Supplementary Figure 12: Forest plot of sensitivity analysis comparing 'age' of patients with large vessel occlusion in the posterior circulation (PC) versus anterior circulation (AC) who were treated with endovascular mechanical thrombectomy; exclusion of studies was based on high risk of selection bias.

Chi^2 = chi-square statistic, CI = confidence interval, df = degrees of freedom, I^2 = I-square heterogeneity statistic, IV = weighted mean difference, P = p value, SD = standard deviation, Std. = standardized, Tau^2 = estimated variance of underlying effects across studies, Z = Z statistic

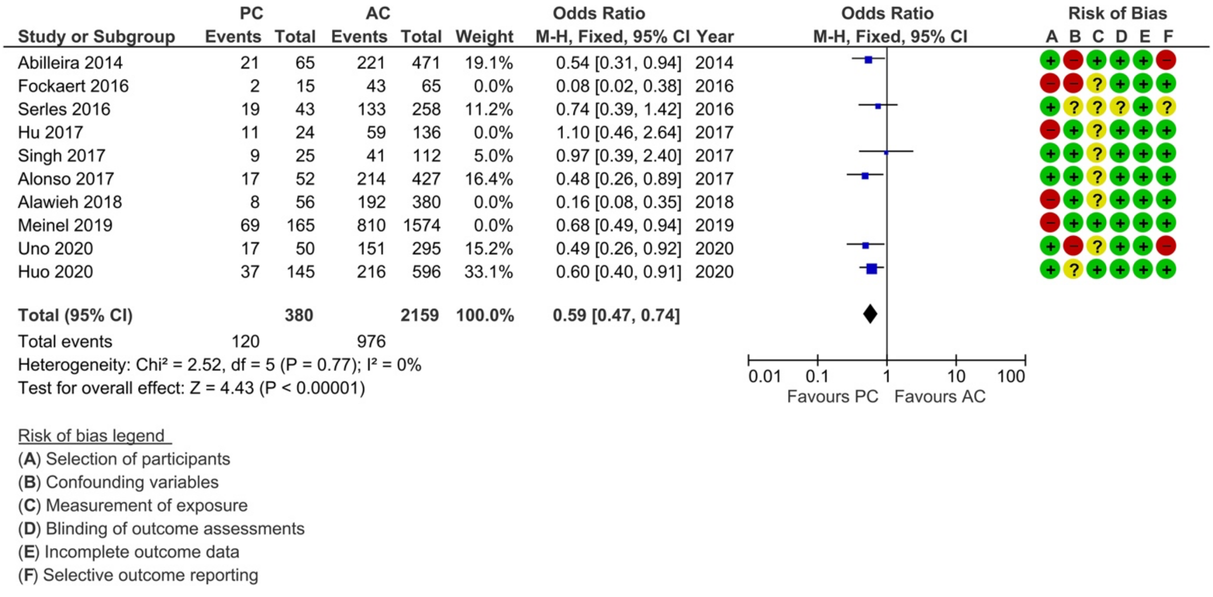

Supplementary Figure 13: Forest plot of sensitivity analysis comparing baseline characteristic 'female sex' of patients with large vessel occlusion in the posterior circulation (PC) versus anterior circulation (AC) who were treated with endovascular mechanical thrombectomy; exclusion of studies was based on high risk of selection bias.

Chi^2 = chi-square statistic, CI = confidence interval, df = degrees of freedom, I^2 = I-square heterogeneity statistic, M-H = Mantel-Haenszel statistic, P = p value, Z = Z statistic

Study or Subgroup	PC Mean	PC SD	PC Total	AC Mean	AC SD	AC Total	Weight	Std. Mean Difference IV, Random, 95% CI
Abilleira 2014	19.43	15.02	65	17.61	5.02	471	14.8%	0.26 [-0.00, 0.52]
Alawieh 2018	17.4	11	56	15.3	7	380	0.0%	0.27 [-0.01, 0.56]
Alonso 2017	19.51	27.23	52	17.61	5.02	427	14.3%	0.19 [-0.10, 0.48]
Fockaert 2016	27.33	25.95	15	46.78	135.1	65	0.0%	-0.16 [-0.72, 0.41]
Hu 2017	25.15	32.38	24	19.01	23.74	136	0.0%	0.24 [-0.19, 0.68]
Huo 2020	23.17	14.08	50	17.67	6.67	295	14.0%	0.67 [0.37, 0.98]
Meinel 2019	18.67	16.31	165	16.33	5.93	1574	0.0%	0.31 [0.15, 0.47]
Mourand 2011	20.67	25.95	15	16.33	10.38	25	0.0%	0.24 [-0.40, 0.88]
Serles 2016	23.48	16.74	43	16.64	5.01	258	13.5%	0.87 [0.54, 1.20]
Singh 2017	19	5.5	25	15.5	4.32	112	11.5%	0.76 [0.32, 1.21]
Uno 2020	19	11.12	145	16.97	6.88	596	16.0%	0.26 [0.08, 0.44]
Weber 2019	15.31	12.72	139	15.97	5.63	961	16.0%	-0.10 [-0.27, 0.08]
**Total (95% CI)**			**519**			**3120**	**100.0%**	**0.39 [0.13, 0.65]**

Heterogeneity: Tau² = 0.10; Chi² = 40.89, df = 6 (P < 0.00001); I² = 85%
Test for overall effect: Z = 2.98 (P = 0.003)

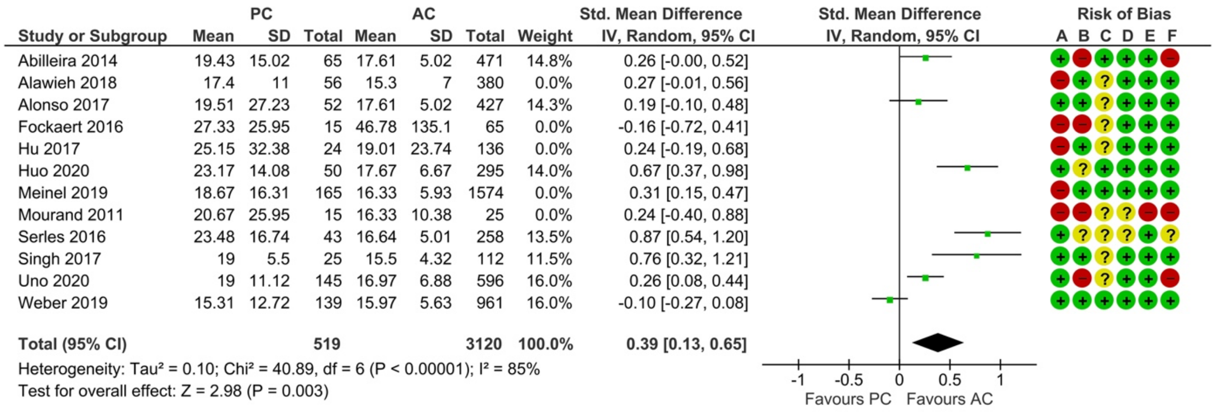

Risk of bias legend
(**A**) Selection of participants
(**B**) Confounding variables
(**C**) Measurement of exposure
(**D**) Blinding of outcome assessments
(**E**) Incomplete outcome data
(**F**) Selective outcome reporting

Supplementary Figure 14: Forest plot of sensitivity analysis comparing 'baseline NIHSS' of patients with large vessel occlusion in the posterior circulation (PC) versus anterior circulation (AC) who were treated with endovascular mechanical thrombectomy; exclusion of studies was based on high risk of selection bias.

Chi^2 = chi-square statistic, CI = confidence interval, df = degrees of freedom, I^2 = I-square heterogeneity statistic, IV = weighted mean difference, P = p value, SD = standard deviation, Std. = standardized, Tau^2 = estimated variance of underlying effects across studies, Z = Z statistic

A

Study or Subgroup	PC Mean	PC SD	PC Total	AC Mean	AC SD	AC Total	Weight	Std. Mean Difference IV, Random, 95% CI	Year
Serles 2016	290.17	77.39	43	256.66	82.6	258	13.9%	0.41 [0.08, 0.73]	2016
Alonso 2017	439.58	187.07	52	342.54	145.19	427	14.3%	0.64 [0.35, 0.94]	2017
Hu 2017	474.36	438.26	24	631.36	794.58	136	12.5%	-0.21 [-0.64, 0.23]	2017
Alawieh 2018	26.3	20	56	28.1	23.5	380	14.4%	-0.08 [-0.36, 0.20]	2018
Weber 2019	427.09	293.34	139	298.64	126.2	961	15.4%	0.82 [0.63, 1.00]	2019
Huo 2020	217.52	83.64	50	244.14	103.5	295	14.2%	-0.26 [-0.56, 0.04]	2020
Uno 2020	436.29	171.48	145	371.16	112.76	596	15.4%	0.51 [0.33, 0.70]	2020
**Total (95% CI)**			**509**			**3053**	**100.0%**	**0.28 [-0.04, 0.60]**	

Heterogeneity: Tau² = 0.16; Chi² = 61.68, df = 6 (P < 0.00001); I² = 90%
Test for overall effect: Z = 1.72 (P = 0.08)

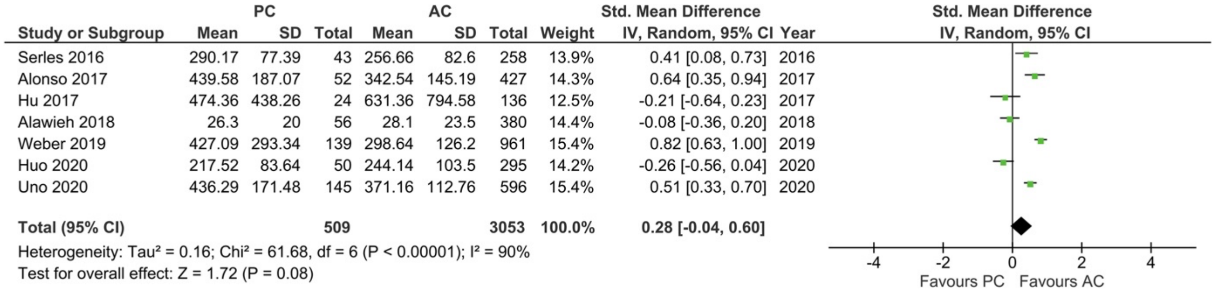

B

Study or Subgroup	PC Mean	PC SD	PC Total	AC Mean	AC SD	AC Total	Weight	Std. Mean Difference IV, Random, 95% CI	Year	Risk of Bias A B C D E F
Serles 2016	290.17	77.39	43	256.66	82.6	258	18.7%	0.41 [0.08, 0.73]	2016	+ ? ? ? + ?
Alonso 2017	439.58	187.07	52	342.54	145.19	427	19.4%	0.64 [0.35, 0.94]	2017	+ + ? + + +
Hu 2017	474.36	438.26	24	631.36	794.58	136	0.0%	-0.21 [-0.64, 0.23]	2017	− + ? + + +
Alawieh 2018	26.3	20	56	28.1	23.5	380	0.0%	-0.08 [-0.36, 0.20]	2018	− + ? + + +
Weber 2019	427.09	293.34	139	298.64	126.2	961	21.3%	0.82 [0.63, 1.00]	2019	+ + + + + +
Huo 2020	217.52	83.64	50	244.14	103.5	295	19.2%	-0.26 [-0.56, 0.04]	2020	+ ? + + + +
Uno 2020	436.29	171.48	145	371.16	112.76	596	21.3%	0.51 [0.33, 0.70]	2020	+ − ? + + −
**Total (95% CI)**			**429**			**2537**	**100.0%**	**0.43 [0.10, 0.77]**		

Heterogeneity: Tau² = 0.13; Chi² = 37.54, df = 4 (P < 0.00001); I² = 89%
Test for overall effect: Z = 2.57 (P = 0.01)

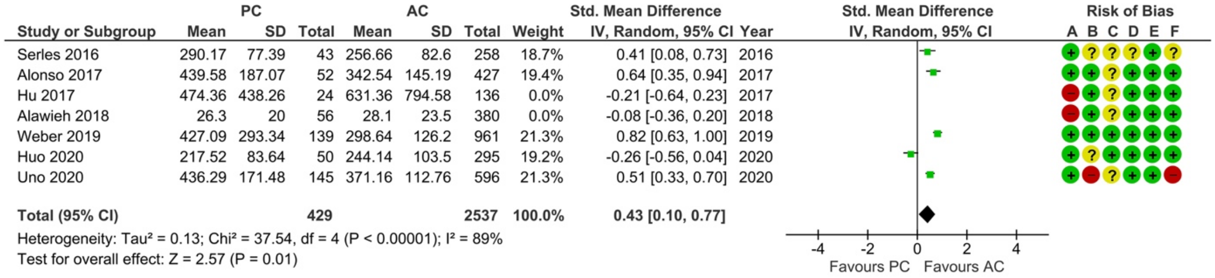

Risk of bias legend
(**A**) Selection of participants
(**B**) Confounding variables
(**C**) Measurement of exposure
(**D**) Blinding of outcome assessments
(**E**) Incomplete outcome data
(**F**) Selective outcome reporting

Supplementary Figure 15: Forest plot comparing 'onset-to-recanalization time' in patients with large vessel occlusion in the posterior circulation (PC) versus anterior circulation (AC) who were treated with endovascular mechanical thrombectomy; A: results for all studies, B: results for sensitivity analysis which excluded studies on the basis of high risk of selection bias.

Chi^2 = chi-square statistic, CI = confidence interval, df = degrees of freedom, I^2 = I-square heterogeneity statistic, IV = weighted mean difference, P = p value, SD = standard deviation, Std. = standardized, Tau^2 = estimated variance of underlying effects across studies, Z = Z statistic

A

Study or Subgroup	PC Mean	PC SD	PC Total	AC Mean	AC SD	AC Total	Weight	Std. Mean Difference IV, Random, 95% CI	Year
Singh 2017	3.39	4.64	52	2	1.48	427	20.3%	0.67 [0.38, 0.96]	2017
Alonso 2017	6.73	19.9	24	16.38	93.81	136	15.3%	-0.11 [-0.54, 0.32]	2017
Hu 2017	1.6	0.65	25	1.54	0.78	112	15.3%	0.08 [-0.35, 0.51]	2017
Weber 2019	2	1.48	139	2	1.48	961	24.3%	0.00 [-0.18, 0.18]	2019
Meinel 2019	2.6	4.28	165	2	1.48	1574	24.8%	0.31 [0.15, 0.47]	2019
**Total (95% CI)**			**405**			**3210**	**100.0%**	**0.21 [-0.05, 0.46]**	

Heterogeneity: Tau² = 0.06; Chi² = 19.02, df = 4 (P = 0.0008); I² = 79%
Test for overall effect: Z = 1.61 (P = 0.11)

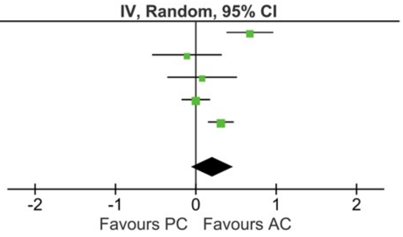

B

Study or Subgroup	PC Mean	PC SD	PC Total	AC Mean	AC SD	AC Total	Weight	Std. Mean Difference IV, Random, 95% CI	Year	Risk of Bias A	B	C	D	E	F
Singh 2017	3.39	4.64	52	2	1.48	427	33.9%	0.67 [0.38, 0.96]	2017	+	+	?	+	+	+
Alonso 2017	6.73	19.9	24	16.38	93.81	136	29.3%	-0.11 [-0.54, 0.32]	2017	+	+	?	+	+	+
Hu 2017	1.6	0.65	25	1.54	0.78	112	0.0%	0.08 [-0.35, 0.51]	2017	–	+	?	+	+	+
Weber 2019	2	1.48	139	2	1.48	961	36.8%	0.00 [-0.18, 0.18]	2019	+	+	+	+	+	+
Meinel 2019	2.6	4.28	165	2	1.48	1574	0.0%	0.31 [0.15, 0.47]	2019	–	+	+	+	+	+
**Total (95% CI)**			**215**			**1524**	**100.0%**	**0.20 [-0.28, 0.67]**							

Heterogeneity: Tau² = 0.15; Chi² = 16.50, df = 2 (P = 0.0003); I² = 88%
Test for overall effect: Z = 0.80 (P = 0.42)

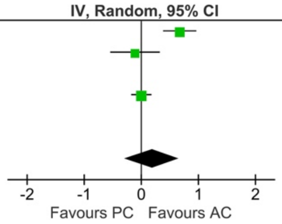

Risk of bias legend
(**A**) Selection of participants
(**B**) Confounding variables
(**C**) Measurement of exposure
(**D**) Blinding of outcome assessments
(**E**) Incomplete outcome data
(**F**) Selective outcome reporting

Supplementary Figure 16: Forest plot comparing ‘number of passages’ for recanalization in patients with large vessel occlusion in the posterior circulation (PC) versus anterior circulation (AC) who were treated with endovascular mechanical thrombectomy; A: results for all studies, B: results for sensitivity analysis which excluded studies on the basis of high risk of selection bias.

Chi^2 = chi-square statistic, CI = confidence interval, df = degrees of freedom, I^2 = I-square heterogeneity statistic, IV = weighted mean difference, P = p value, SD = standard deviation, Std. = standardized, Tau^2 = estimated variance of underlying effects across studies, Z = Z statistic

Study or Subgroup	PC Events	PC Total	AC Events	AC Total	Weight	Odds Ratio M-H, Random, 95% CI	Year	A	B	C	D	E	F
Abilleira 2014	24	65	261	471	15.4%	0.47 [0.28, 0.80]	2014	+	−	+	+	+	−
Fockaert 2016	6	15	39	65	0.0%	0.44 [0.14, 1.40]	2016	−	−	?	+	+	+
Serles 2016	31	43	162	258	11.0%	1.53 [0.75, 3.12]	2016	+	?	?	?	+	?
Hu 2017	9	24	64	136	0.0%	0.68 [0.28, 1.65]	2017	−	+	?	+	+	+
Singh 2017	7	25	62	112	7.4%	0.31 [0.12, 0.81]	2017	+	+	?	+	+	+
Alonso 2017	20	52	220	427	13.9%	0.59 [0.33, 1.06]	2017	+	+	?	+	+	+
Alawieh 2018	19	56	153	380	0.0%	0.76 [0.42, 1.37]	2018	−	+	?	+	+	+
Meinel 2019	71	165	779	1574	0.0%	0.77 [0.56, 1.07]	2019	−	+	+	+	+	+
Weber 2019	58	139	505	961	21.2%	0.65 [0.45, 0.93]	2019	+	+	+	+	+	+
Huo 2020	25	145	187	596	17.6%	0.46 [0.29, 0.72]	2020	+	?	+	+	+	+
Uno 2020	26	50	170	295	13.6%	0.80 [0.44, 1.45]	2020	+	−	?	+	+	−
**Total (95% CI)**		**519**		**3120**	**100.0%**	**0.61 [0.46, 0.82]**							
Total events	191		1567										

Heterogeneity: Tau² = 0.07; Chi² = 11.58, df = 6 (P = 0.07); I² = 48%
Test for overall effect: Z = 3.25 (P = 0.001)

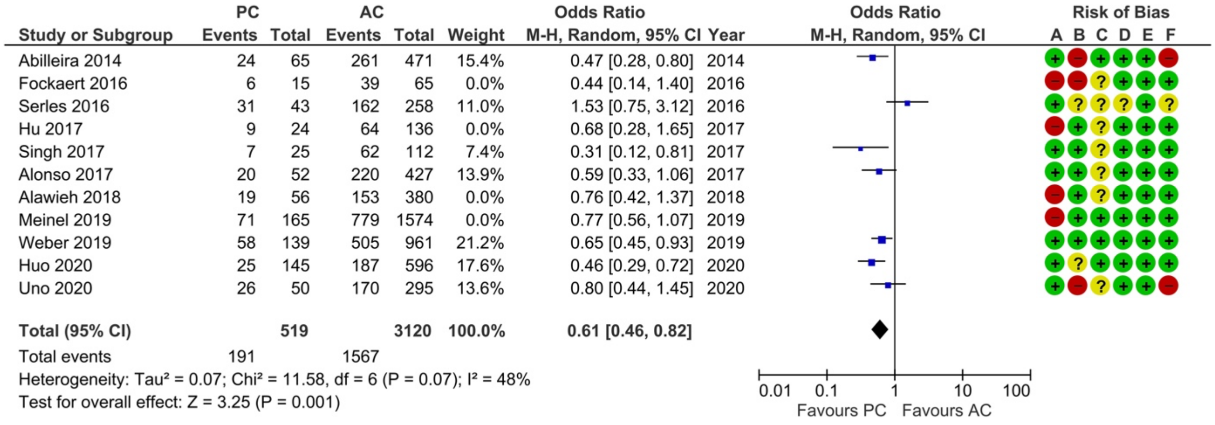

Risk of bias legend
(**A**) Selection of participants
(**B**) Confounding variables
(**C**) Measurement of exposure
(**D**) Blinding of outcome assessments
(**E**) Incomplete outcome data
(**F**) Selective outcome reporting

Supplementary Figure 17: Forest plot of sensitivity analysis comparing rate of intravenous thrombolysis in patients with large vessel occlusion in the posterior circulation (PC) versus anterior circulation (AC) who were treated with endovascular mechanical thrombectomy; exclusion of studies was based on high risk of selection bias.

Chi^2 = chi-square statistic, CI = confidence interval, df = degrees of freedom, I^2 = I-square heterogeneity statistic, M-H = Mantel-Haenszel statistic, P = p value, Tau^2 = estimated variance of underlying effects across studies, Z = Z statistic

Study or Subgroup	PC Mean	PC SD	PC Total	AC Mean	AC SD	AC Total	Weight	Std. Mean Difference IV, Random, 95% CI	Year	A	B	C	D	E	F
Serles 2016	158.2	64.64	43	104.67	36.32	258	49.0%	1.29 [0.95, 1.63]	2016	+	?	?	?	+	?
Alonso 2017	152.49	44.02	52	124.84	41.47	427	51.0%	0.66 [0.37, 0.95]	2017	+	+	?	+	+	+
Meinel 2019	170.05	72.38	39	129.64	62.73	437	0.0%	0.63 [0.30, 0.96]	2019	−	+	+	+	+	+
**Total (95% CI)**			**95**			**685**	**100.0%**	**0.97 [0.35, 1.58]**							

Heterogeneity: Tau² = 0.17; Chi² = 7.54, df = 1 (P = 0.006); I² = 87%
Test for overall effect: Z = 3.09 (P = 0.002)

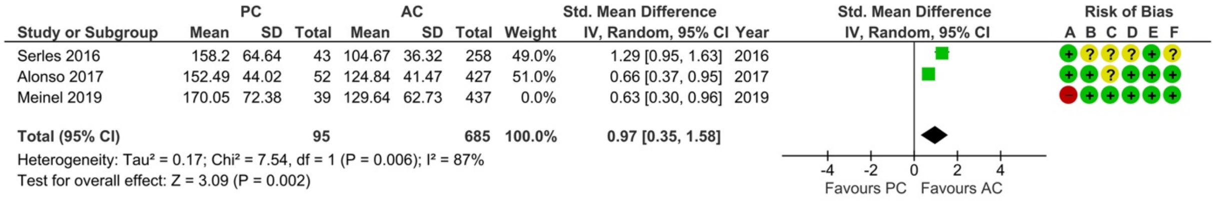

Risk of bias legend
(**A**) Selection of participants
(**B**) Confounding variables
(**C**) Measurement of exposure
(**D**) Blinding of outcome assessments
(**E**) Incomplete outcome data
(**F**) Selective outcome reporting

Supplementary Figure 18: Forest plot of sensitivity analysis comparing 'onset-to-intravenous thrombolysis time' in patients with large vessel occlusion in the posterior circulation (PC) versus anterior circulation (AC) who were treated with endovascular mechanical thrombectomy; exclusion of studies was based on high risk of selection bias.

Chi^2 = chi-square statistic, CI = confidence interval, df = degrees of freedom, I^2 = I-square heterogeneity statistic, IV = weighted mean difference, P = p value, SD = standard deviation, Std. = standardized, Tau^2 = estimated variance of underlying effects across studies, Z = Z statistic

Study or Subgroup	PC Mean	PC SD	PC Total	AC Mean	AC SD	AC Total	Weight	Std. Mean Difference IV, Random, 95% CI	Year
Abilleira 2014	464.8	386.75	65	291.43	137.1	471	25.0%	0.93 [0.67, 1.20]	2014
Serles 2016	251.87	83.13	43	199.05	74.31	258	21.3%	0.70 [0.37, 1.02]	2016
Alonso 2017	402.39	286.48	52	302.18	126.22	427	23.5%	0.66 [0.37, 0.95]	2017
Alawieh 2018	480.3	845.6	56	491.7	812.5	380	0.0%	-0.01 [-0.29, 0.27]	2018
Meinel 2019	379.86	266.93	165	254.99	130.87	1574	0.0%	0.84 [0.67, 1.00]	2019
Huo 2020	347.22	155.39	145	286.28	135.76	596	30.2%	0.44 [0.25, 0.62]	2020
**Total (95% CI)**			**305**			**1752**	**100.0%**	**0.67 [0.44, 0.90]**	

Heterogeneity: Tau² = 0.04; Chi² = 9.52, df = 3 (P = 0.02); I² = 68%
Test for overall effect: Z = 5.64 (P < 0.00001)

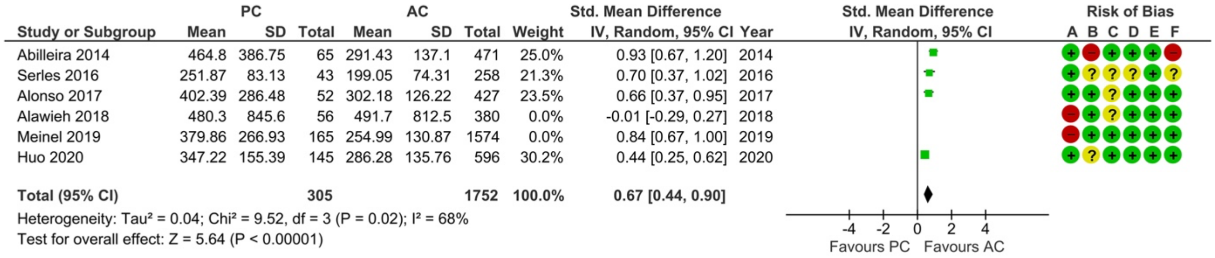

Risk of bias legend
(**A**) Selection of participants
(**B**) Confounding variables
(**C**) Measurement of exposure
(**D**) Blinding of outcome assessments
(**E**) Incomplete outcome data
(**F**) Selective outcome reporting

Supplementary Figure 19: Forest plot of sensitivity analysis comparing 'onset-to-groin puncture time' in patients with large vessel occlusion in the posterior circulation (PC) versus anterior circulation (AC) who were treated with endovascular mechanical thrombectomy; exclusion of studies was based on high risk of selection bias.

Chi^2 = chi-square statistic, CI = confidence interval, df = degrees of freedom, I^2 = I-square heterogeneity statistic, IV = weighted mean difference, P = p value, SD = standard deviation, Std. = standardized, Tau^2 = estimated variance of underlying effects across studies, Z = Z statistic

Supplementary Tables

Supplementary Table 1: Comorbidities reported in included studies

	Posterior circulation								Anterior circulation							
Publication	N	HT	AF	HL	Smoking	Diabetes	CAD	Prior stroke or TIA	N	HT	AF	HL	Smoking	Diabetes	CAD	Prior stroke or TIA
Mourand 2011	15	–	–	–	–	–	–	–	25	–	–	–	–	–	–	–
Abilleira 2014	65	37	13	22	15	17	13	16	471	284	145	169	87	93	56	58
Lefevre 2014	26	–	–	–	–	–	–	–	36	–	–	–	–	–	–	–
Fockaert 2016	15	–	4	–	–	–	–	–	65	–	29	–	–	–	–	–
Serles 2016	43	–	–	–	–	–	–	–	258	–	–	–	–	–	–	–
Alonso 2017	52	27	13	20	19	8	4	6	427	268	175	193	96	81	60	42
Hu 2017	24	8	13	7	–	9	5	1	136	58	84	37	–	42	65	4
Khoury 2017	5	–	–	–	–	–	–	–	35	–	–	–	–	–	–	–
Singh 2017	25	11	1	–	6	11	1	–	112	56	5	–	28	24	12	–
Alawieh 2018	56	15	40	15	–	11	–	–	380	148	261	152	–	110	–	–
Meinel 2019	165	87 [*]		60 [**]	47 [***]	23 [#]	17 [##]	17 [###]	1,574	1,046 [‡]	–	788 [‡‡]	428 [‡‡‡]	271 [§]	235 [§§]	209 [§§§]
Weber 2019	139	94	42	44	30	19	–	26	961	745	411	346	142	191		152
Wollenweber 2019	303	–	–	–	–	–	–	–	2,265	–	–	–	–	–	–	–
Uno 2020	50	26	10	14	11	7	3	12	295	184	76	73	70	59	43	64
Renieri 2020	44	–	–	–	–	–	–	–	90	–	–	–	–	–	–	–
Huo 2020	145	98	20	–	49	27	–	19	596	298	135	–	195	85	–	58
Total		403	156	182	177	132	43	97		3,087	1,321	1,758	1,046	956	471	587
Percentage (%)		56.1	27.3	33.3	28.1	18.4	14.4	15.3		62.6	38.4	41.7	24.0	19.4	18.3	13.2

[*] n = 163, [**] n = 161, [***] n = 153, [#] n = 163, [##] n = 83, [###] n = 159, [‡] n = 1,553, [‡‡] n = 1,547, [‡‡‡] n = 1,499, [§] n = 1,555, [§§] n = 1,131, [§§§] n = 1,565; – = not available, AF = atrial fibrillation, CAD = coronary artery disease, HL = hyperlipidemia, HT = hypertension, N = number of patients, n = reference number of patients, TIA = transient ischemic attack

Supplementary Table 2: Etiology of large vessel occlusion reported in included studies

Publication	Posterior circulation					Anterior circulation				
	N	Large artery atherosclerosis	Cardiac embolism	Other determined cause	Cryptogenic	N	Large artery atherosclerosis	Cardiac embolism	Other determined cause	Cryptogenic
Mourand 2011	15	–	–	–	–	25	–	–	–	–
Abilleira 2014	65	27	23	6	5	471	89	239	25	85
Lefevre 2014	26	–	–	–	–	36	–	–	–	–
Fockaert 2016	15	0	6	6	7	65	6	38	4	17
Serles 2016	43	–	18	–	–	258	–	114	–	–
Alonso 2017	52	10	18	6	17	427	64	234	23	98
Hu 2017	24	–	–	–	–	136	–	–	–	–
Khoury 2017	5	–	–	–	–	35	–	–	–	–
Singh 2017	25	–	–	–	–	112	–	–	–	–
Alawieh 2018	56	–	–	–	–	380	–	–	–	–
Meinel 2019	163	37	64	12	50	1,546	205	733	101	507
Weber 2019	139	45	46	15	31	961	231	458	41	196
Wollenweber 2019	303	–	–	–	–	2,265	–	–	–	–
Uno 2020	50	10	38	–	2	295	44	236	6	3
Renieri 2020	44	–	–	–	–	90	–	–	–	–
Huo 2020	145	102	21	22	–	596	368	121	107	–
Total		231	234	67	112		1,007	2,173	301	906
Percentage (%)		36.7	34.8	11.6	23.1		23.1	47.0	7.0	24.1

– = not available, N = number of patients

Supplementary References

Kim, S.Y., Park, J.E., Lee, Y.J., Seo, H.J., Sheen, S.S., Hahn, S., Jang, B.H., and Son, H.J. (2013). Testing a tool for assessing the risk of bias for nonrandomized studies showed moderate reliability and promising validity. *J Clin Epidemiol* 66**,** 408-414.
